# The detailed analysis of the microbiome and resistome of artisanal blue-veined cheeses provides evidence on sources and patterns of succession linked with quality and safety traits

**DOI:** 10.1186/s40168-024-01790-4

**Published:** 2024-04-27

**Authors:** Elena A. Alexa, José F. Cobo-Díaz, Erica Renes, Tom F. O´Callaghan, Kieran Kilcawley, David Mannion, Iwona Skibinska, Lorena Ruiz, Abelardo Margolles, Paula Fernández-Gómez, Adrián Alvarez-Molina, Paula Puente-Gómez, Fiona Crispie, Mercedes López, Miguel Prieto, Paul D. Cotter, Avelino Alvarez-Ordóñez

**Affiliations:** 1 Department of Food Hygiene and Technology, Universidad de León, León, Spain; 2 School of Food and Nutritional Sciences, University College Cork, Cork, T12 Y337 Ireland; 3 Teagasc Food Research Centre, Fermoy, Co., Cork Ireland; 4 Dairy Research Institute, Spanish National Research Council, Instituto de Productos Lácteos de Asturias-CSIC, Villaviciosa, Spain; 5 Functionality and Ecology of Beneficial Microbes (MicroHealth) Group, Instituto de Investigación Sanitaria del Principado de Asturias (ISPA), 33011 Oviedo, Asturias Spain; 6 Institute of Food Science and Technology, Universidad de León, León, Spain; 7 APC Microbiome Ireland, University College Cork, Cork, Ireland; 8 VistaMilk, Cork, Ireland

**Keywords:** Cheese, Microbiome, Processing environments, Source tracking

## Abstract

**Background:**

Artisanal cheeses usually contain a highly diverse microbial community which can significantly impact their quality and safety. Here, we describe a detailed longitudinal study assessing the impact of ripening in three natural caves on the microbiome and resistome succession across three different producers of *Cabrales* blue-veined cheese.

**Results:**

Both the producer and cave in which cheeses were ripened significantly influenced the cheese microbiome. *Lactococcus* and the former *Lactobacillus* genus, among other taxa, showed high abundance in cheeses at initial stages of ripening, either coming from the raw material, starter culture used, and/or the environment of processing plants. Along cheese ripening in caves, these taxa were displaced by other bacteria, such as *Tetragenococcus*, *Corynebacterium*, *Brevibacterium*, *Yaniella*, and *Staphylococcus*, predominantly originating from cave environments (mainly food contact surfaces), as demonstrated by source-tracking analysis, strain analysis at read level, and the characterization of 613 metagenome-assembled genomes. The high abundance of *Tetragenococcus koreensis* and *Tetragenococcus halophilus* detected in cheese has not been found previously in cheese metagenomes. Furthermore, *Tetragenococcus* showed a high level of horizontal gene transfer with other members of the cheese microbiome, mainly with *Lactococcus* and *Staphylococcus*, involving genes related to carbohydrate metabolism functions. The resistome analysis revealed that raw milk and the associated processing environments are a rich reservoir of antimicrobial resistance determinants, mainly associated with resistance to aminoglycosides, tetracyclines, and *β*-lactam antibiotics and harbored by aerobic gram-negative bacteria of high relevance from a safety point of view, such as *Escherichia coli*, *Salmonella enterica*, *Acinetobacter*, and *Klebsiella pneumoniae*, and that the displacement of most raw milk-associated taxa by cave-associated taxa during ripening gave rise to a significant decrease in the load of ARGs and, therefore, to a safer end product.

**Conclusion:**

Overall, the cave environments represented an important source of non-starter microorganisms which may play a relevant role in the quality and safety of the end products. Among them, we have identified novel taxa and taxa not previously regarded as being dominant components of the cheese microbiome (*Tetragenococcus* spp.), providing very valuable information for the authentication of this protected designation of origin artisanal cheese.

Video Abstract

**Supplementary Information:**

The online version contains supplementary material available at 10.1186/s40168-024-01790-4.

## Background

Artisanal cheeses, including those which are recognized in the EU with protected designation of origin (PDO), are highly valuable premium products due to their unique sensory attributes relative to mass-produced cheeses. Their quality, shelf life, and safety are dependent on a number of factors but are especially influenced by the diverse microbial communities they harbor, which vary from the core to the rind of the cheese and evolve throughout the cheese-making and ripening process [1, 2]. Moreover, they can reflect differences among producers or regions in a manner that can be applied to verify the authenticity of PDO products [3] and can act as carriers of antimicrobial resistance genes (ARGs) and other hazards that may spread to humans and pose a safety risk [4].

Despite the substantial efforts devoted to study microbial succession patterns during the production and ripening of a wide variety of cheeses, certain aspects relating to microbial interactions and their functional implications remain largely unknown [5]. Historically, the microbiome of artisanal cheeses has been characterized using culture-based methods, which have several limitations [3, 5]. High-throughput sequencing methodologies (e.g., metataxonomy or whole metagenome sequencing) have transformed the way we study microbial ecology in complex ecosystems, such as those of artisanal cheeses [3, 6–9], as they provide access to difficult-to-culture or viable but not culturable microorganisms [1, 7] and facilitate the mechanistic understanding of community assembly in such a way that allows studying temporal dynamics of microbial communities at an unprecedented level of detail [7]. Some previous studies have highlighted that cheese and other fermented foods can serve as useful models for elucidating the determinants of microbial succession and interactions in complex communities, including understanding the molecular processes and key metabolically active microbes that contribute to the development of unique sensorial characteristics or that impact the product safety [7, 8, 10]. In the present work, the microbiome and resistome of *Cabrales* cheese, a PDO artisanal Spanish blue-veined cheese, were deeply characterized. *Cabrales* cheese is produced in the mountainous area of “Picos de Europa” (*Principado de Asturias*, northern Spain) from raw cow, sheep, and/or goat milk. This cheese is ripened for up to 5 months in natural caves above 800-m altitude, with > 90% humidity and a temperature ranging from 6 to 10 °C, where it develops its unique intense flavor, creaminess texture, and blue-veins from the rind to the core [11]. The unique microenvironments in these natural caves can shape the microbiome and resistome of the end products promoting the establishment in the cheese of specific bacteria, yeasts, and molds that can impart distinctive quality attributes to the ripened blue-veined cheeses and impact their safety, particularly in relation to the spread of ARGs. To understand these complex phenomena, we undertook an in-depth longitudinal study investigating the structure and functional potential of microbial communities of cheeses (cores and rinds) during the ripening process through whole metagenome sequencing. This has allowed to obtain an insight into the relationships between traditional cheese-making environments and microorganisms prevailing in cheese and potentially affecting quality and safety.

## Results

### General patterns of microbial succession

The experimental setup included cheeses from three different producers who used the same cave for ripening. In addition, for one producer (Producer2), cheeses derived from a single production batch were ripened in three different caves, in order to identify microbiome signatures associated with the respective cave microenvironments (Fig. S1).

Regardless of producer or cave used for ripening, cheeses maintained certain shared characteristics and microbial succession patterns. Counts of total aerobic bacteria, lactic acid bacteria, Enterobacteriaceae, and yeasts and molds reached maximum levels at initial ripening stages (Stage1) and then gradually declined during ripening in the caves (Stage2 and Stage3), both for cheese cores and rinds (Fig. S2A). Culture-independent analysis revealed that at Stage1, cheeses were dominated by lactic acid bacteria, mainly *Lactococcus* and members of the former *Lactobacillus* genus, and by the yeast *Debaryomyces* and the fungi *Penicillium* and *Geotrichum* (Fig. S2B). Whereas fungal populations remained fairly stable along ripening, a significant temporal decrease in the relative abundance of *Lactococcus* and *Lactobacillus*, among others, was evident (Fig. S2B), while other taxa, such as *Tetragenococcus*, *Brevibacterium*, and *Corynebacterium*, emerged with high relative abundances at Stage2 and Stage3, especially at rind level (Fig. S2B).

### Microbiome differences by producer

**Fig. 1:**
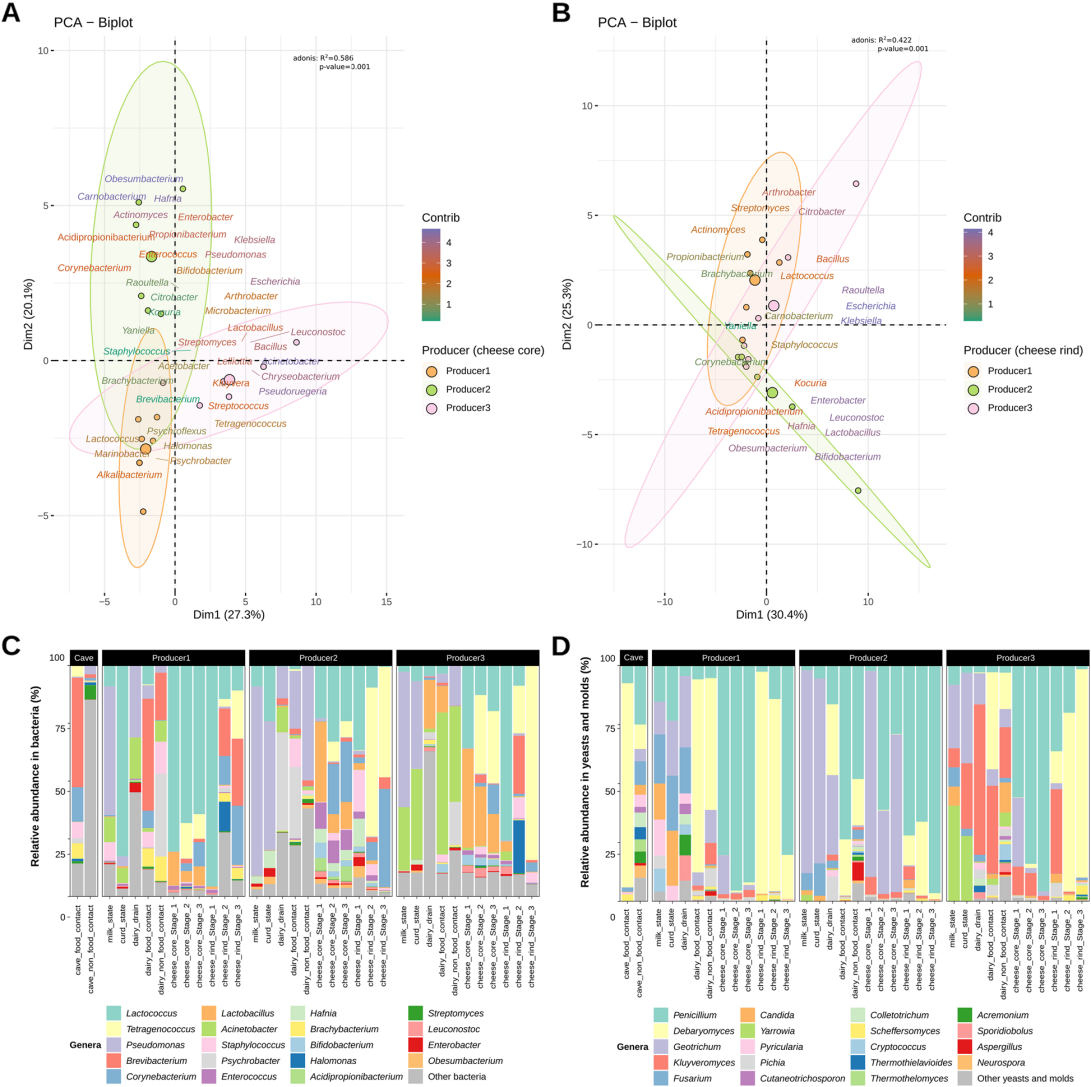
Microbiome of blue-veined cheeses during ripening in the communal cave. Biplots of the principal component analysis of the 40 most abundant bacterial genera found in the cheese core (**A**) and cheese rind (**B**) samples ripened in the communal cave depending on the producer. The biplots show the most significant bacterial genera contributing to the microbiome of cheeses ripened in Cave2 (communal cave) by the three producers. Colored concentration ellipses (size determined by a 0.95-probability level) show the observations grouped by producer. Barplots representing the relative abundance of the 19 most relevant bacterial (**C**) and yeasts and molds (**D**) genera. Other minority bacterial and fungal genera are grouped into “other.” Each bar represents the average value (*n* = 3) for blue-veined cheese samples from each producer

The cheese producer had a significant influence on the bacterial and fungal taxonomic profile of samples (Fig. 1A, B, Fig. S3A, B), both for cheese cores (adonis, *p* = 0.001 for both bacteria and fungi) and rinds (adonis, *p* = 0.001 for bacteria and *p* = 0.018 for fungi). At cheese core level, several bacterial genera within the main ones such as *Corynebacterium*, *Enterococcus*, among others were associated with Producer2, while *Lactobacillus* and *Tetragenococcus* were predominantly linked to Producer3 and *Lactococcus* to Producer1 (Fig. 1A, C, Table S1). At cheese rind level, some of the main bacterial genera were clearly associated with Producer2 (e.g., *Corynebacterium*), while *Lactococcus*, *Brevibacterium*, and *Brachybacterium* were associated with Producer1 and *Tetragenococcus* and *Staphylococcus* with Producer3 (Fig. 1B, C, Table S1). Regarding fungi, core samples showed a high abundance of *Penicillium*, but, for Producer2, similar relative abundances of *Geotrichum* and *Penicillium* were observed. At rind level, *Penicillium* and *Debaryomyces* showed high abundances in samples from Producer1 and Producer2, while *Debaryomyces* was the dominant genus for Producer3 (Fig. 1D, Table S1).

*Lactobacillus* includes all the newly established genera that formerly belonged to the genus *Lactobacillus* (e.g., *Lacticaseibacillus*, *Lactiplantibacillus*).

### Microbiome differences by ripening cave

**Fig. 2:**
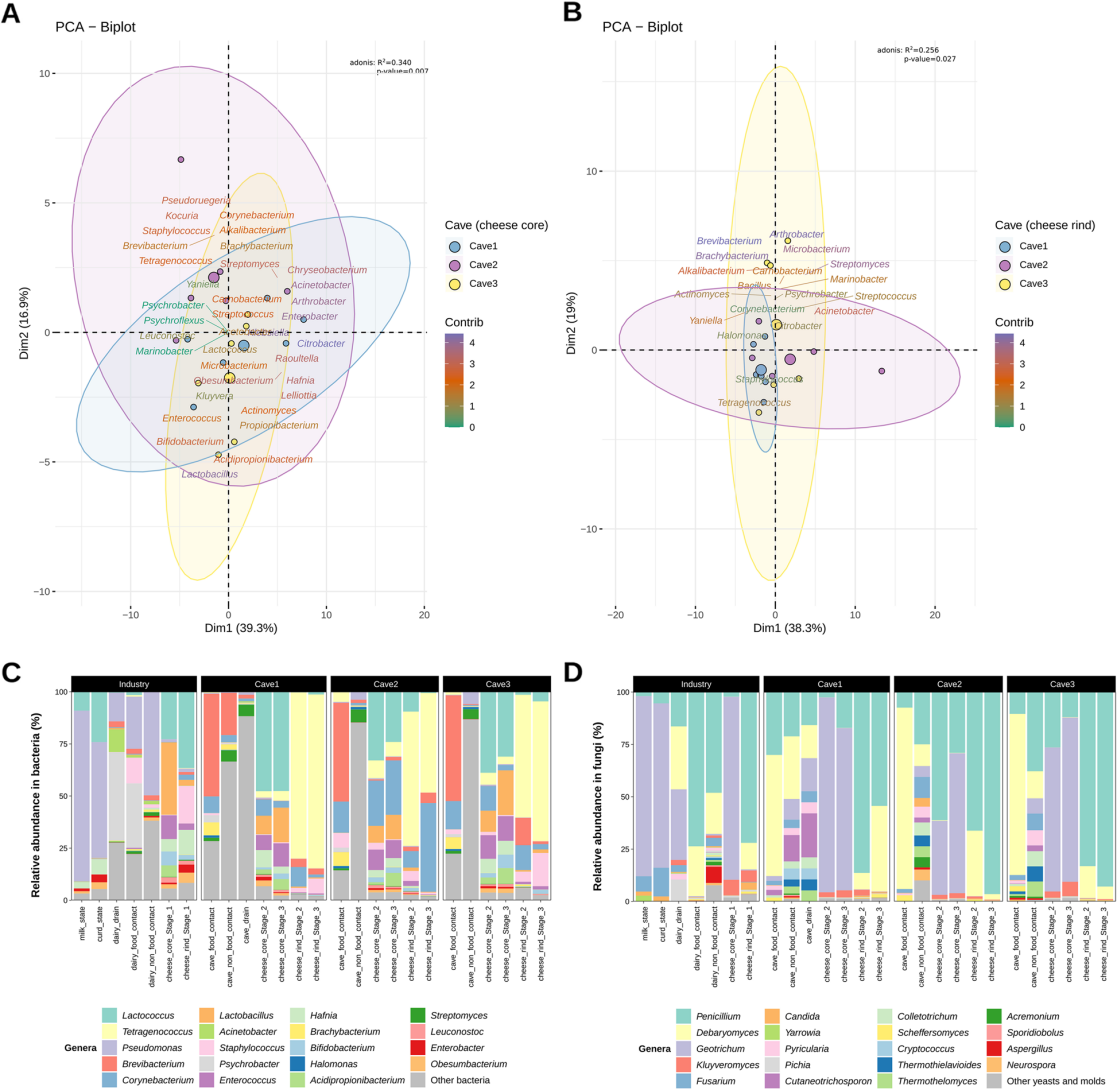
Microbiome of blue-veined cheeses from Producer2 during ripening in the three different caves. Biplot of the principal component analysis of the 40 most abundant bacterial genera found in the cheese core (**A**) and cheese rind (**B**) samples ripened in the three different caves. The biplots show the most significant bacterial genera contributing to the cheese microbiome in the three different caves. Colored concentration ellipses (size determined by a 0.95-probability level) show the observations grouped by cave. Barplots representing the relative abundance of the 19 most relevant bacterial (**C**) and yeasts and molds (**D**) genera. Other minority bacterial and fungal genera are grouped into “other.” Each bar represents the average value (*n* = 3) for blue-veined cheese samples in each cave. *Lactobacillus* includes all the newly established genera that formerly belonged to the genus *Lactobacillus* (e.g., *Lacticaseibacillus*, *Lactiplantibacillus*)

The cave also had a significant influence, although lower than that found for the variable Producer, on the taxonomic profile of cheese cores (adonis, *p* = 0.007 for bacteria and *p* = 0.049 for fungi) and rinds (adonis, *p* = 0.027 for bacteria and 0.653 for fungi) (Fig. 2A, B, Fig. S3C, D). The most relevant differences among caves in the abundance of some particular taxa were those observed for *Lactococcus* and the former *Lactobacillus*, which were more abundant on core samples from Cave1 and Cave3, respectively, at Stage3; *Tetragenococcus*, *Corynebacterium*, and *Staphylococcus*, which were more abundant on rind samples from Cave1, Cave2, and Cave3, respectively (Fig. 2A, C, Table S2); and *Debaryomyces*, which was significantly more abundant on cheese rinds from Cave1 than on those from the other two caves (Fig. 2D, Table S2).

### The cave microbiome strongly shapes the rind cheese microbiome

The characterization of the microbial communities prevailing in some primary sources that could determine the cheese microbiome (e.g., milk, curd, different plant processing environments and cave environments) showed that raw milk was dominated by *Pseudomonas*, followed by *Acinetobacter*, *Staphylococcus*, or *Lactococcus*, at different levels depending on the producer (Fig. S4). Similar profiles but with a higher abundance of some lactic acid bacteria, such as *Lactococcus*, were found in curd samples (Fig. S4). *Brevibacterium*, *Psychrobacter*, *Pseudomonas*, *Acinetobacter*, *Penicillium*, *Debaryomyces*, and *Kluyveromyces* were the dominant genera on food processing environments (both in food contact and nonfood contact samples) of the three cheese-producing plants (Fig. S4). *Brevibacterium*, *Corynebacterium*, and *Debaryomyces* were the dominant genera in cave food contact surfaces, while *Penicillium* and a wide range of nondominant bacterial and fungal genera prevailed on the caves’ nonfood contact surfaces (Fig. S4).

**Fig. 3:**
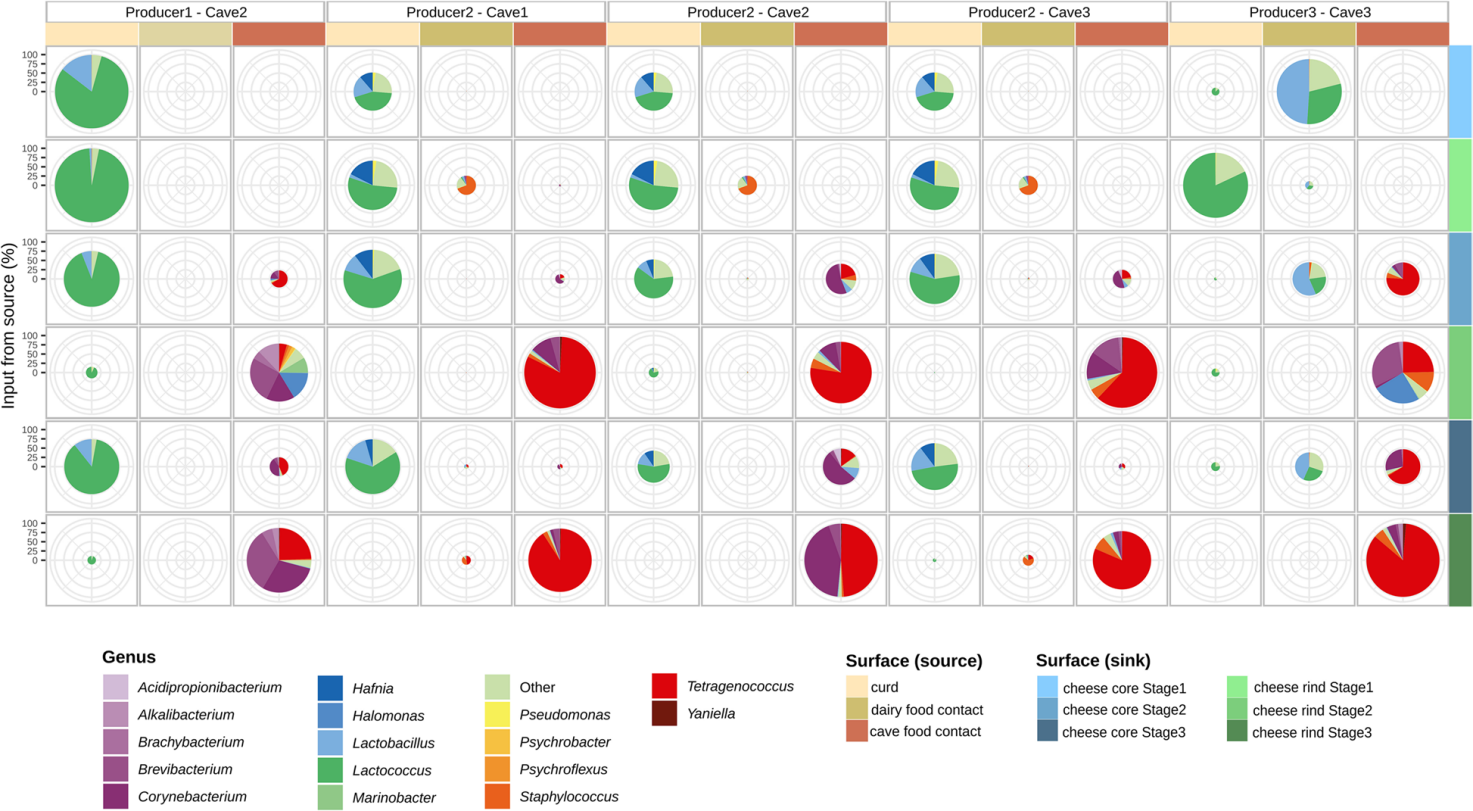
Taxonomic (bacteria) source attribution of cheese samples calculated by SourceTracker2 software. Pie chart plots represent bacterial sources (on columns, *source* samples) for the cheese bacterial community composition (on rows, *sink* samples). The ratio of the pie chart is proportional to the percentage of *source* sample influence on *sink* sample (indicated on the *y*-axis). The colors within each pie chart indicate the percentage of genus influence for each source-sink pair. Only *source* samples and the 16 main genera with significant influence were represented, while other genera were grouped as “Other.” *Lactobacillus* includes all the newly established genera that formerly belonged to the genus *Lactobacillus* (e.g., *Lacticaseibacillus*, *Lactiplantibacillus*)

A source-tracking analysis revealed that the bacterial composition of cheese cores, and of cheese rinds at Stage1, was mainly determined, for Producer1 and Producer2, by the curd microbiota, and for Producer3 by the microbiota of food contact environments from the processing plant (Fig. 3). The main bacterial genera traced back to the curd and dairy food contact surfaces were *Lactococcus*, the former *Lactobacillus*, and, in the case of Producer2, *Hafnia* (Fig. 3). Over subsequent stages of ripening, the curd microbiota still represented an important source for the cheese core microbiome, although the cave environment had an important role in shaping the microbiome of cheese cores on Cave2 at Stage2 and Stage3, mainly being a source of *Tetragenococcus* and *Corynebacterium* for Producer1 and Producer3, and *Corynebacterium* for Producer2 (Fig. 3). Other less abundant genera (e.g., *Brachybacterium*, *Alkalibacterium*, *Staphylococcus*) were also traced back to the cave environments. Regarding cheese rinds, their bacterial microbiota at Stage2 and Stage3 were traced back mainly to the cave environments, which were revealed as a likely source of *Brevibacterium*, *Corynebacterium*, and *Tetragenococcus* (on Stage3) and other minority genera (on Stage2), for Producer1; *Tetragenococcus* in Cave1 and Cave3, together with *Corynebacterium* in Cave2, for Producer2; and *Tetragenococcus*, *Brevibacterium* and other minority genera (on Stage2), and *Tetragenococcus* (on Stage3) for Producer3 (Fig. 3).

A source-tracking analysis undertaken on the fungal communities showed that food contact surfaces from Cave2 were a source of *Debaryomyces* in rind samples from Producer1 and Producer3, and milk was a source of *Geotrichum* in core samples from Producer2 (Fig. S5).

### Strain-level analyses confirmed the relevance of caves as a source of bacteria

**Fig. 4:**
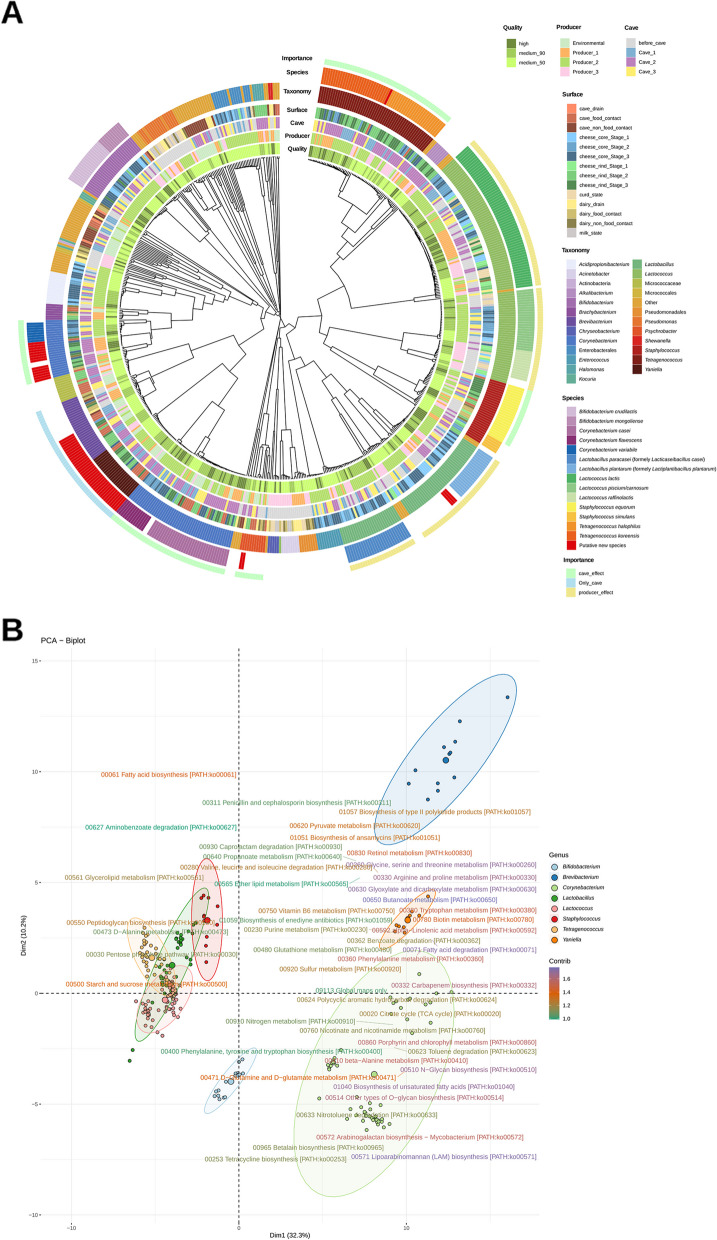
Phylogenetic tree of MAGs obtained by per-sample assembly and binning of metagenomic reads. **A** Phylogenetic tree constructed with the ANI distance matrix generated by using the dRep software. Concentric circles indicate, from inside to outside, the quality of MAGs; producer, cave, and surface origin of the sample; genus and species classification of the MAGs; and importance of the MAGs. Only the 24 most abundant taxa at genus level (or above) are indicated, while the rest are grouped in “Other.” Some taxa are classified in higher taxonomic levels (phylum, family, or order) according to the deep level of taxonomic assignment obtained by using the CAT/BAT software. Indicated species were taxonomically reassigned by building additional phylogenetic trees employing reference genomes (RefSeq database) or other representative genomes from the NCBI. The last circle indicates clusters of interest for cave and producer effects on the microbiome. Only new species within the genera of interest (*Brevibacterium*, *Corynebacterium*, *Lactobacillus*, *Tetragenococcus*, and *Yaniella*) were marked with red color on the “species” circle. **B** Principal coordinate analysis of the MAGs functional composition by level 3 of KEGG Orthology classification. Only functional groups belonging to *09100 Metabolism* were employed. Only MAGs of interest due to their importance related to the producer and/or cave influence were employed. *Lactobacillus* includes all the newly established genera that formerly belonged to the genus *Lactobacillus* (e.g., *Lacticaseibacillus*, *Lactiplantibacillus*)

A total of 110 high-quality metagenome-assembled genomes (MAGs), 227 medium quality MAGs with completeness > 90%, and 276 medium-quality MAGs with completeness of 50–90% were obtained. They were classified into 70 genera, with the main genera represented being *Lactococcus* (103 MAGs), *Corynebacterium* (74 MAGs), the former *Lactobacillus* (68 MAGs), *Tetragenococcus* (54 MAGs), *Bifidobacterium* (30 MAGs), *Staphylococcus* (28 MAGs), *Brevibacterium* (28 MAGs), and *Yaniella* (22 MAGs). Cheese samples were the ones with the highest number of MAGs (413 out of 613 MAGs), while raw materials, processing environments at factory level, and cave environments yielded 43, 74, and 83 MAGs, respectively (Fig. 4A, Fig. S6).

MAGs from several putative new species were obtained on samples from cave surfaces or cheeses. These included 14 *Brevibacterium* sp. MAGs most closely related to *Brevibacterium sandarakinum*, 8 *Corynebacterium* sp. MAGs related to *Corynebacterium nuruki*, 6 *Corynebacterium* sp. MAGs related to *Corynebacterium glyciniphilum*, 2 *Psychrobacter* sp. MAGs, and 1 *Tetragenococcus* sp. MAG closely related to *T. koreensis*. Remarkably, despite the low relative abundance of *Yaniella* found at read level, a total of 22 MAGs from this genus, closely related to the recently discovered *Candidatus Yaniella excrementavium*, were obtained (Fig. 4A, Fig. S6). Furthermore, three MAGs assigned to the former genus *Lactobacillus*, obtained from cheese core samples from Producer3 at Stage1, were not assigned at species level, with *Lactobacillus selangorensis* and *Lactobacillus camelliae* being the most closely related species (Fig. 4A).

It is worth noting that MAGs assigned to *Lactococcus*, a dominant genus at Stage1, presented phylogenetic differences by producer, with *Lactococcus lactis* MAGs from Producer2 clustering separately from MAGs from other producers, which was also observed in a strain-level StrainPhlAn population genomics analysis (Fig. S7). Moreover, *Lactococcus piscium/carnosum* MAGs were exclusively found for Producer2 and *Lactococcus raffinolactis* and *Lactococcus garvieae* MAGs for Producer3 (Fig. 4A). On the contrary, the phylogenetic tree corresponding to the strain-level StrainPhlAn population genomics analysis detected *L. raffinolactis* on both Producer2 and Producer3, with two clear clusters (Fig. S7). On the other hand, different closely related MAGs from *Corynebacterium casei*, *Staphylococcus equorum*, *Brevibacterium* sp., *Tetragenococcus halophilus*, *T. koreensis*, and *Yaniella* sp. were obtained from both cheese samples and cave environments. Moreover, in the case of cheese MAGs from *Brevibacterium*, *Tetragenococcus*, and *Yaniella*, they were only obtained from cheeses at Stage2 and Stage3 (Fig. 4A, Fig. S6, Fig. S7). Similar results were observed in the strain-level StrainPhlAn population genomics analyses, with the absence of these genera at Stage1, while they were within the main ones found at Stage2 and Stage3 (Fig. S7). Moreover, a clear influence of the cave was observed at the species level of reads taxonomic assignment, where cheese rinds from Producer2 had *T. koreensis* as the dominant species on ripened cheeses from Cave3, while *T. halophilus* dominated for Cave1 and Cave2 (Fig. S8). It was also interesting to see that two different clusters of *S. equorum*, with differentiated functional potential, were detected colonizing food processing environments within factories and cheeses at Stage1 and cave environments and cheeses at Stage2 and Stage3, respectively (Fig. 4A, Fig. S9).

The comparison of the MAGs from *T. koreensis* and *T. halophilus* with the available genomes from the NCBI showed a clear separation of *T. koreensis* genomes from PDO *Cabrales* (current study) and *Picón Bejes-Tresviso* cheese [12] from others from nondairy food isolates (thick juice, soy sauce, fermented or salted seafood, fermented vegetables), with similar observations for *T. halophilus* (Fig. S10).

The analysis of the functional potential of MAGs evidenced that *Lactococcus*, *Lactobacillus*, *Staphylococcus*, and *Tetragenococcus* had a relatively similar functional profile, which was characterized by the high abundance of functions related to the metabolism of carbohydrates, while *Corynebacterium*, *Brevibacterium*, and *Yaniella* MAGs had a very different functional profile, characterized by the high abundance of different functions mainly related to protein and fatty acids metabolism (Fig. 4B).

### The cheese microbiome is rich in horizontal gene transfer events

**Fig. 5:**
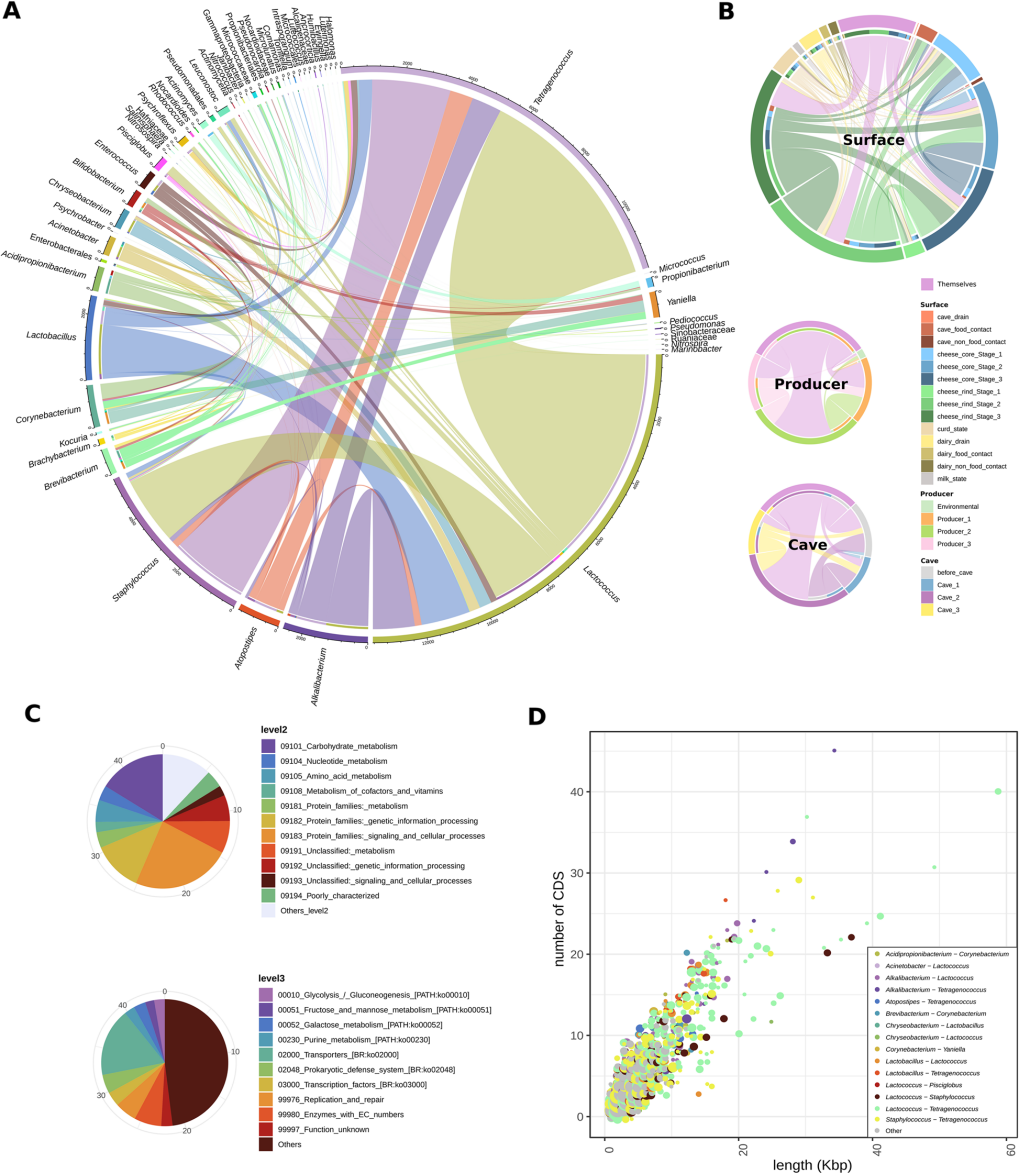
Horizontal gene transfer (HGT) events. HGT events (identity > 99.9%, length > 500 bp) detected between MAGs belonging to different genera (**A**) and classified by surface, producer, and cave (**B**). The thickness of the circle internal lines is proportional to the number of HGT events detected for each genera pair. The “Themselves” group corresponds with HGT events between different genera from the same surface, producer, or cave group. **C** Percentage of coding regions (CDS) detected within HGT sequences belonging to different functions of the level 2 and level 3 of KEGG Orthology functional classification. Only 45% of CDS detected, with KO code assigned, were represented. **D** Relation between the length of HGT events and the number of CDS detected in them. Circle colors indicate the genera pair for each HGT event, while the size of the circles is square-root proportional to the number of HGT events with the same length and CDS number. *Lactobacillus* includes all the newly established genera that formerly belonged to the genus *Lactobacillus* (e.g., *Lacticaseibacillus*, *Lactiplantibacillus*)

In order to identify among the main bacterial taxa clues of microbiome acclimatization to cave and cheese microenvironments, signs of horizontal gene transfer (HGT) events were searched in the available MAGs. A total of 23,001 HGT events, containing 67,411 coding regions, were detected between 56 taxa above species level, with *Lactococcus*, *Tetragenococcus*, and *Staphylococcus* being the main genera involved (59.3, 52.0, and 23.3% of total HGT events, respectively) and *Lactococcus*-*Tetragenococcus*, *Staphylococcus*-*Tetragenococcus*, and *Lactococcus*-*Staphylococcus* as the main HGT pairs (29.6, 10.9, and 9.4% of total HGT events, respectively) (Fig. 5A). HGT events were identified from MAGs belonging to all surfaces sampled, but cheese core and rind samples at Stages 2 and 3 of ripening contributed the most (56.6% of HGT events) (Fig. 5B).

Up to 35.3% of the HGT events were associated with plasmid sequences, according to PlasFlow analysis, while only 0.5% carried relaxase encoding genes, associated with plasmid mobilization. Moreover, 11.7, 8.1, and 0.9% harbored prophages, transposases, and integrases, respectively. Although 55.5% of the coding regions could not be assigned to ko codes of the KEGG Orthology (KO) database, 10.6, 7.2, and 5.4% were assigned at level 2 to the groups *protein families: signaling and cellular processes*, *carbohydrate metabolism*, and *protein families: genetic information processing*, respectively. At level 3 of KO classification, *prokaryotic defense system*, *galactose metabolism*, *glycolysis/gluconeogenesis*, *purine metabolism*, and *fructose and mannose metabolism* were within the dominant functions (Fig. 5C). Finally, 68 HGT-associated contigs were longer than 20 kb, containing from 12 to 45 coding regions (CDS) (Fig. 5D), and clustered into 26 groups. *Lactococcus*-*Tetragenococcus* and *Alkalibacterium*-*Tetragenococcus* were the dominant pairs within these longest HGT events, with 14 and 4 clusters, respectively (Table S3). Half of the obtained HGT clusters contained genes related to mobile genetic elements such as plasmids, transposases, phages, and integrons; three clusters harbored genes related to resistance to β-lactam antibiotics, and up to nine clusters contained genes related to protein, carbohydrate, or lipid metabolism (Table S3). Furthermore, a blastn search against the ResFinder database, using only the HGT sequences, rendered only 10 positive hits (identity and coverage > 80%), which were associated to heat, β-lactams, and tetracycline resistance, being *L. lactis*, *T. koreensis*, and *Staphylococcus equorum* the main species found in these ARG-related HGT events (Table S4).

### Resistome evolution throughout the cheese-making and ripening process

**Fig. 6:**
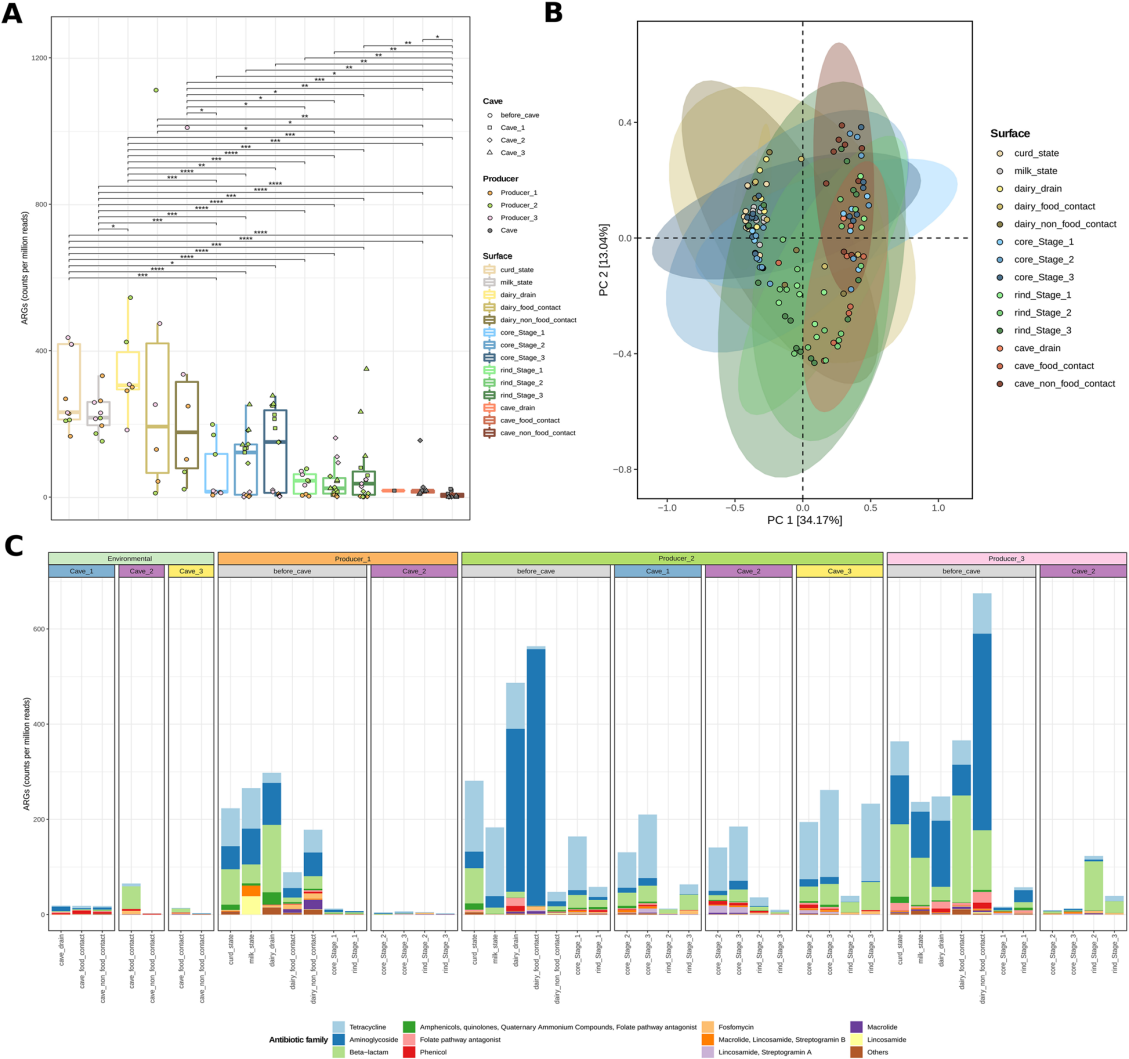
Evolution of the resistome of blue-veined cheeses along ripening. Boxplot of total ARGs detected, expressed in counts per million reads (**A**). Only significant differences (*p*-value < 0.05) are indicated. Boxplot color indicates surface, jitter plot color indicates producer, and the shape indicates the cave. Principal component analysis of the resistome, considering ARGs classified by antibiotic family, grouped by surface (**B**). Barplots representing the relative abundance of the 11 most relevant ARG families (**C**). Other minority antibiotic families are grouped into “Others.” Each bar represents the average value for blue-veined cheese samples (*n* = 3) and environmental samples (*n* = 2)

The total load of antimicrobial resistance genes (ARG) (excluding genes related to resistance against *heat* and *quaternary ammonium compounds*) was higher on raw materials and processing environments of the cheese production plant than in the rest of samples (cheeses and cave environments). Moreover, cheese rinds presented lower ARG loads than cheese core samples. Cheeses from Producer2 had higher ARG loads than cheeses from other producers at core level, while showed low ARG loads in rind samples, except for cheeses from Cave3 (Fig. 6A). A PCoA analysis, performed at antibiotic family level and not considering *heat* and *quaternary ammonium compounds* resistance gene families, yielded two main sample clusters with differentiated resistome profile, the first one containing samples from raw materials, plant processing environments, and cheese core samples and the second cluster mainly with cheese rind samples at Stage2 and Stage3 and samples from cave environments (adonis: *p*-value = 0.001, Fig. 6B). Furthermore, the variable Producer had a stronger influence on ordination of samples according to ARG family composition than the variable Cave, as it can be observed when comparing PCoA analyses performed with samples from Cave2, originating from different producers (adonis *p*-value = 0.001), or with samples from Producer2, ripened in different caves (adonis *p*-value = 0.078) (Fig. S11). The most abundant ARGs detected were those associated with resistance to beta-lactams, aminoglycosides and tetracyclines in samples from food processing environments and raw materials, tetracyclines in cheese samples from Producer2, and beta-lactams in cheese samples from Producer3 (Fig. 6C).

**Fig. 7:**
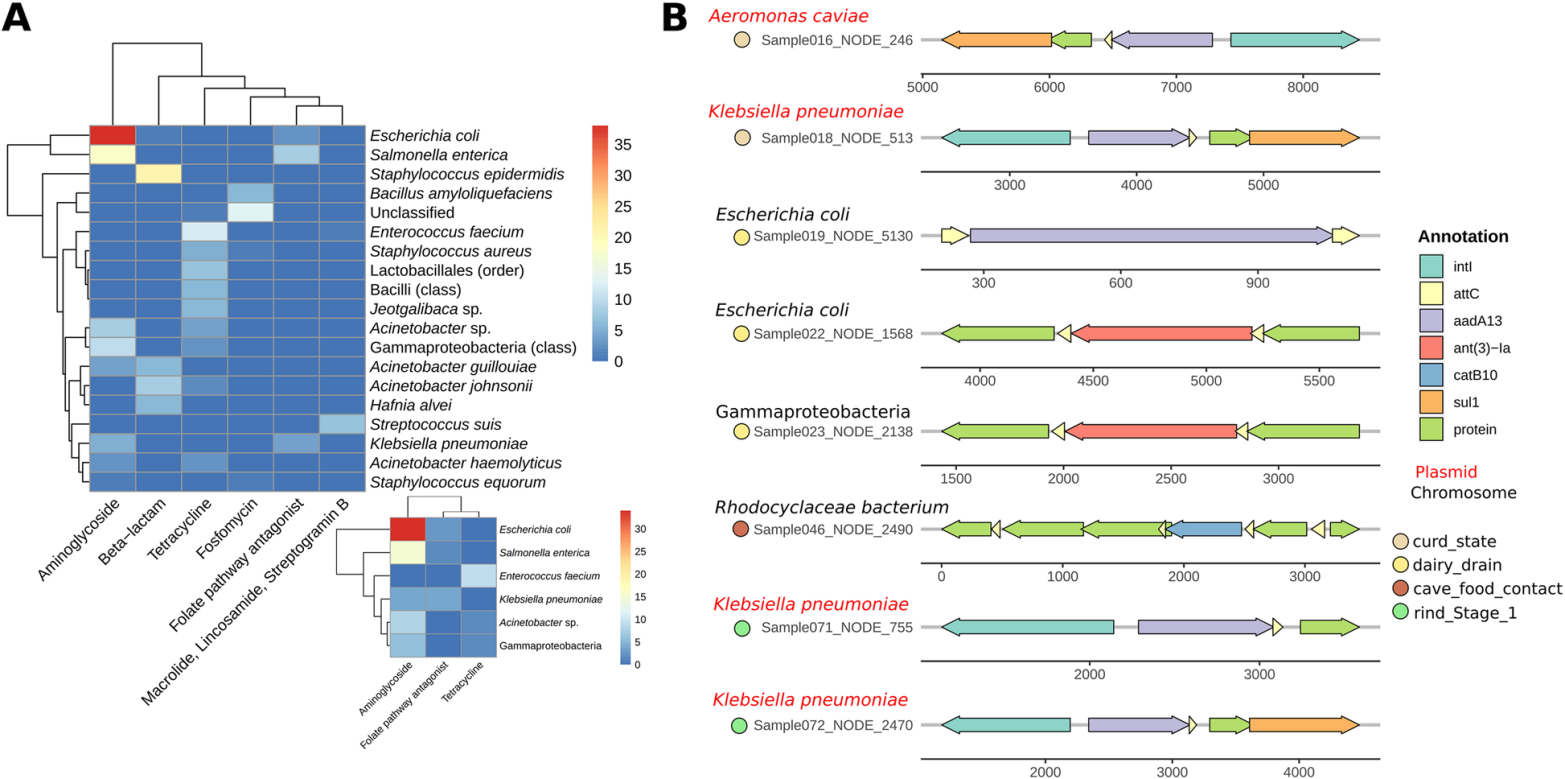
Assembly-based analysis of the resistome and its association with taxa and mobile genetic elements. Heatmaps representing the number of contigs found harboring ARGs related to the antibiotic families indicated on columns, while the rows indicate the taxonomic assignment of contigs by Kraken2 (**A**). The small heatmap represents only those ARG-carrying contigs classified as plasmidic contigs according to the Platon pipeline. Only those antibiotic families and taxa with five or more contigs are included. Schematic representation of all the ARG-carrying integrons found (**B**). Arrow colors indicate the presence of the integrase gene (“intI”), “attc” regions, ARGs (ARG name indicated), or genes coding for proteins not assigned as ARG (indicated as “protein”). Text color of taxonomical assignment (performed by Kraken2) indicates if the contig is plasmidic or chromosomic, according to the Platon pipeline. Circle colors indicate the type of sample from where the contig was obtained

Assembly-based analyses allowed the identification of a total of 317 ARG-carrying contigs, containing up to 350 different ARGs. Up to 222 of these ARGs are associated with resistance to 6 antibiotic families, with aminoglycosides, tetracyclines, and beta-lactams as the most abundant (87, 49, and 42 ARG-carrying contigs, respectively). ARG-carrying contigs were most frequently assigned to *Escherichia coli*, *Salmonella enterica*, and *Staphylococcus epidermidis* (42, 26, and 21 contigs, respectively), harboring ARGs mainly associated with resistance to aminoglycosides for *E. coli* and *S. enterica*, and to beta-lactams for *S. epidermidis* (Fig. 7A). Remarkably, many ARG-carrying contigs were classified as plasmidic and associated with well-known pathogenic species, such as *Acinetobacter*, *Enterococcus faecium*, *E. coli*, *Klebsiella pneumoniae*, and *S. enterica*, mainly from the ESKAPEE group (Fig. 7A). It is also worth noting the identification of ARGs associated with resistance to aminoglycosides (*aadA13*) and folate pathway antagonists (*sul1*) within integrons located on contigs classified as plasmidic, mainly assigned to *K. pneumoniae* and coming from curd samples and cheeses at Stage1 (before entering in the caves) (Fig. 7B).

## Discussion

Our study found that two different groups of microorganisms prevailed in *Cabrales* PDO cheese. The first group comprises various taxa (e.g., *Lactococcus*, the former *Lactobacillus*, *Bifidobacterium*, *Penicillium*, *Geotrichum*, and *Debaryomyces*) that show a high abundance in the cheeses from the initial stages of production, prior to the cheeses entering the ripening caves. These microorganisms very likely originate from the raw materials, the starter cultures used, and/or the dairy plant processing environments. This group of microbes, with the exception of *Debaryomyces*, occurred at high abundances in cheese cores over the whole ripening period, showing in some cases intraspecific phylogenetic diversity depending on the cheese producer. However, as ripening progressed within the cave, this group of microbes was gradually displaced, especially on cheese rinds, by the second group of microbes, which comprises several taxa (e.g., *Tetragenococcus*, *Corynebacterium*, *Brevibacterium*, *Yaniella*, *S. equorum*) not found (or present at very low abundance) in milk, curd, or environments within the processing plant and cheeses at the initial stages of ripening. These microbes are very likely sourced within the cave environments, as evidenced by SourceTracker predictions and the close genomic relationships among some MAGs from cheese and cave samples, such as some representatives of *S. equorum*, *T. koreensis*, *T. halophilus*, and *C. casei*.

The role of some members of the *Cabrales* cheese microbiome in the ripening process is evident based on previous studies for this sort of cheeses. Indeed, *Lactococcus*, *Lactobacillus*, *Staphylococcus*, *Corynebacterium*, *Brevibacterium, Penicillium*, *Geotrichum*, or *Debaryomyces* have all previously been detected at high abundance in blue-veined rind washed cheeses, and in some cases also in cheese processing environments [6, 7, 13], with very clear contributions to cheese ripening. Moreover, some of these taxa, such as *Corynebacterium* or *Brevibacterium*, play a particularly important role in aroma development during cheese ripening [6, 13, 14].

On the other hand, other taxa of the *Cabrales* cheese microbiome have received much less attention in the past or have been detected at high relative abundance in cheese in our study for the first time. Specifically, this is the case of one low abundant species of *Yaniella* and two species from *Tetragenococcus* (*T. koreensis* and *T. halophilus*), found in very high abundance in the latest stages of *Cabrales* cheese ripening. Although the presence of *Yaniella* in food-related environments has been recently described [2, 15], its relevance during cheese ripening remains unclear. Here, we describe a total of 22 *Yaniella* sp. MAGs (8 of them from cave environments), closely related to the recently discovered *Candidatus Yaniella excrementavium*, for which further research is needed to understand its contribution to the quality and safety of *Cabrales* cheese. *Tetragenococcus* has been isolated from Brie cheese [16], two Mexican cheeses [17], and, very recently, from two Spanish traditional blue-veined cheeses [12]. *T. koreensis* strains have been very recently isolated from Cabrales cheese, while we are reporting the occurrence of both *T. koreensis* and *T. halophilus*, with different core-rind distribution, in the current study. The two *Tetragenococcus* species here detected have a functional profile similar to that of some traditional lactic acid bacteria (e.g., *Lactococcus*, *Lactobacillus*) and possibly displace them due to their better acclimatization to the microenvironments associated with our study. *Tetragenococcus* are halo-alkaliphilic bacteria, and the relatively high salt content and progressive increase in pH along ripening, especially at rind level (data not shown), can provide ideal conditions to promote their growth [18]. Remarkably, the dominance of one of the two *Tetragenococcus* species over the other depended on both the producer and ripening cave, and when both *Tetragenococcus* species co-occurred, *T. halophilus*, with a higher optimum pH for growth of 6.1–7.0 [16], showed tropism for the cheese rind and *T. koreensis*, with an optimum pH for growth of 5.7–6.5 [16], for the cheese core. The identification of these taxa in a very high relative abundance in cheeses from a very restricted geographical region suggests that they may be used as microbiome markers for PDO authentication purposes.

Moreover, the results provide evidence of high levels of horizontal gene transfer among bacteria belonging to different genera, suggesting that these events can mediate strain adaptation to the cheese/cave microenvironments, as has been previously proposed for cheese rind microbiomes [19]. A relatively high proportion of such HGT-associated sequences harbored mobile genetic elements. Likewise, high levels of phage and transposase-associated genes have been previously found on MAGs obtained from Swiss Gruyère cheese [20]. Remarkably, extensive HGT was observed for *Tetragenococcus* with *Lactococcus* and *Staphylococcus*, and many associated genes were related to carbohydrate metabolism functions (e.g., galactose metabolism, glycolysis/gluconeogenesis, and fructose and mannose metabolism). The high diversity of lactose metabolism genes and operons previously found in *Tetragenococcus* [12, 16] and the presence of mobilization elements and sequences with high nucleotide identity to *Streptococcus* and *Staphylococcus* surrounding those loci suggest the likely acquisition by *Tetragenococcus* through HGT of these clusters from other species from the dairy environment [16], considering also the high frequency of HGT events in cheese microbiomes [19].

Our study also evidences that raw milk and the associated processing environments are a rich reservoir of antimicrobial resistance determinants, mainly associated with resistance to aminoglycosides, tetracyclines, and β-lactam antibiotics and harbored by aerobic gram-negative bacteria of high relevance from a safety point of view, such as *E. coli*, *S. enterica*, *Acinetobacter*, and *K. pneumoniae*, in agreement with findings from previous studies [21, 22]. Antimicrobial-resistant bacterial communities of *Cabrales* cheese were previously assessed, and *E. coli*, *Enterococcus faecalis*, and *Staphylococcus* sp. were the taxa identified as those more frequently associated with tetracycline and erythromycin resistance [23]. Moreover, plasmids harboring determinants of resistance to these antimicrobials have been also characterized [24]. Likewise, a high prevalence of class 1 integrons and determinants of resistance to aminoglycosides, tetracyclines, and β-lactam antibiotics have been reported in Brazilian white soft cheeses [25]. Remarkably, the microbial successions taking place in our study during cheese ripening, with the gradual reduction of Enterobactericeae counts and the displacement of most raw milk-associated taxa by cave-associated taxa, gave rise to a significant decrease in the load of ARGs and, therefore, to a safer end product. Indeed, most taxa sourced within the cave environments and dominating the cheese rind microbiome (e.g., *Tetragenococcus*, *Corynebacterium*, *Brevibacterium*) were not identified as major carriers of ARGs. For instance, while Rodríguez et al. isolated from *Cabrales* cheese *Tetragenococcus* strains which showed resistance to erythromycin and clindamycin, no resistance determinants were assigned to this genus in our study [12]. Remarkably, this beneficial effect of ripening was more evident in cheeses ripened in Cave2, especially in those from Producer1 and Producer3, which shows that the resistome is shaped both by the initial microbiome composition of the manufactured cheeses and by the microenvironments prevailing during ripening.

## Conclusions

Overall, our study highlights that cave environments represent an important source of non-starter microorganisms which very likely play a relevant role in the ripening of artisanal blue-veined cheeses and impact their quality and safety and identifies among them various novel taxa and taxa not previously regarded as being dominant components of the cheese microbiome (*Tetragenococcus* spp.), providing very valuable information to the authentication of this PDO artisanal cheese.

## Materials and methods

### Cheese production, sampling strategy, and sample collection

*Cabrales* cheese is the most popular traditional blue-veined cheese in Spain and has a protected designation of origin (PDO) status since 1981. This cheese is produced in “Picos de Europa” (a region in northern Spain) from raw cow, sheep, or goat milk or a mixture of two or all three types of milk. Producers 1 and 3 from our study employed cow milk, while Producer2 used a mixture of cow-sheep-goat milk (88, 8, and 4%, respectively) for cheese production. The traditional cheese-making process of *Cabrales* cheese involves curdling mixtures of evening and morning milk at 28–30 °C using rennet exclusively of animal origin. During cheese making, raw milk is inoculated with a lyophilized lactic acid bacteria starter culture, comprising various strains of *L. lactis*, and a liquid suspension of spores from a strain of *Penicillium roqueforti*. Curds are cut to hazelnut grain size and placed in cylindrical molds at room temperature, being turned upside down several times in order to drain them off without applying pressure. Then, dry salt is added to the cheese surface, and cheeses are taken to a ripening chamber where they remain for around 15 days at 70–80% RH and 8–12 °C. Once the first stage of ripening within the factory is finished, cheeses are placed in natural caves, which are characterized for being deep, with a north-facing entry, and for having at least two openings with flowing water in order to create an airstream. These conditions, complemented by the cave altitude (160, 700, and 1000 m above sea level for Cave1, Cave2, and Cave3 in our study, respectively), lead to very high RH (98, 92–93, and 95% RH for Cave1, Cave2, and Cave3, respectively), with temperatures ranging from 8 to 14 °C (14, 12, and 8–9 °C for Cave1, Cave2, and Cave3, respectively). Cheeses remain in the caves for 2 to 5 months, and during this time, they are periodically turned upside down and washed (with tap water in Cave1 and with running water in Cave2 and Cave3) and brushed twice per month in Cave1 and Cave2 and once per month in Cave3. As a result, influenced by the cave environment, *Cabrales* cheese develops its unique quality characteristics [11].

In each of the three cheese processing facilities, the following samples were taken for analysis: 200 mL of milk and curd samples (three replicates) during the cheese-making process, swab samples from the processing environments entering in contact with the cheese (e.g., work tables, cheese vat, cheese molds), swab samples from non-food contact surfaces (e.g., drains, floors, walls), and three cheeses (2.0 kg each) taken 30 days after cheese making, just before leaving the factory to the ripening cave/s.

In the natural caves, the following samples were taken: swab environmental samples (food contact and non-food contact surfaces, including drains if available) and three cheeses (2.0 kg each cheese) for each of the producers at both the intermediate (90 to 120 days of ripening) and final (130 to 190 days of ripening) ripening stages.

Environmental samples were collected by swabbing using sterile sponges pre-moistened with 10 mL of neutralizing buffer, which were placed individually in sterile bags (3 M, MN, USA).

Appropriate personal protective equipment and gloves were used during all samplings to avoid cross-contamination. At each collection point, the samples were placed in a cooling box containing ice packs and transported to the laboratory within 2 to 3 h. A detailed description of the samples taken is provided in Fig. S1.

Upon arrival at the lab, the cheese rinds were scraped with a sterile knife until the cheese paste was visible (≃0.7 cm). A different sterile knife was used to prepare cheese core samples in order to avoid any possible cross contamination from the rind.

### Culture-based microbiological analyses

For microbiological analyses, 10 g of cheese (rind and core, separately), milk, or curd was homogenized with 90 mL of Buffered Peptone Water (BPW; Merck, Germany) for 2 min in a Stomacher 400 lab blender (Seward Medical, London, UK), while 10 mL of BPW was directly added to the sterile bags containing swab environmental samples. Serial decimal dilutions were then prepared in BPW and spread in triplicate on the following media: (i) plate count agar (PCA; Pronadisa, Spain) for mesophilic bacteria, incubated for 24 ± 2 h at 37 °C; (ii) De Man-Rogosa-Sharpe agar (MRS; Merck) for lactic acid bacteria (LAB), incubated for 72 ± 2 h at 30 °C; (iii) oxytetracycline-glucose-yeast extract agar (OGYEA; Pronadisa) for yeasts and molds, incubated for 5 days at 25 °C; and (iv) violet red bile glucose agar (VRBGA; Pronadisa) for Enterobacteriaceae, incubated for 24 ± 2 h at 37 °C. The results were expressed as means and standard deviations of log_10_ CFU (colony-forming units) per gram from three independent replicates.

### Whole metagenome sequencing analyses

#### DNA extraction

Prior to DNA extraction, curd, cheese, and milk samples were prepared by homogenizing 10 g or mL in 90 mL of BPW and centrifuging at 6500 g for 8 min. Then, pellets were washed with 50 mL of BPW, followed by a new centrifugation at 6500 g for 8 min to harvest the associated microbiota. For the environmental samples, pools of five swabs from each sample category were mixed with 10 mL of BPW. After thorough homogenization, the BPW solutions were centrifuged at 6500 g for 8 min. Pellets from all samples were kept at − 80 °C until further use. Total metagenomic DNA was extracted from the pellets by using the DNeasy PowerSoil Pro Kit (QIAGEN GmbH, Germany) following the manufacturer’s instructions, but conducting a double elution step with 25 µL of 10-mM Tris–HCl, in order to improve DNA yields.

### Library construction and shotgun sequencing

Extracted DNA was employed to prepare 150-bp paired-end sequencing libraries using the Illumina Nextera XT Library Preparation Kit (Illumina Inc., San Diego, CA, USA). Sequencing was performed on the Illumina NextSeq 500 platform using a NextSeq 500/550 High Output Reagent kit v2 (300 cycles), in accordance with the standard Illumina sequencing protocols.

### Reads quality filtering and annotation

Quality filtering of raw reads was performed with AfterQC v0.9.6 [26] using default parameters. Reads matching to cow and sheep genomes were removed using a combination of bowtie2 v2.3.4.1 [27], samtools v1.9 [28], and bedtools v2.29.0 (https://bedtools.readthedocs.io/en/latest/#) according to a pipeline previously published (http://www.metagenomics.wiki/tools/short-read/remove-host-sequences). The taxonomic assignment of filtered reads was done using Kraken2 v2.0.8-beta [29] with the maxikraken2-1903_140GB database (https://lomanlab.github.io/mockcommunity/mc_databases.html) for bacterial taxonomy, while a custom database with kraken2-microbial database plus 20 reference fungal genomes from species found often in cheese environments (Table S5) was employed for fungal taxonomy assignation, due to the absence of major fungal species on the maxikraken2-1903_140GB database.

### Reads assembly and MAGs binning

Each sample was independently subjected to de novo metagenomic assembly through metaSPAdes v3.13 [30] using default parameters. Filtered reads were mapped against contigs higher than 1000 bp obtained from the same sample using bowtie2 v2.3.4.1 [27] with the *–very-sensitive-local* parameter. The *jgi_summarize_bam_contig_depths* script, from MetaBAT2 v2.12.1 [31], was used to calculate contigs depth values, mandatory for per-sample contig binning based on tetranucleotide frequency and the contig abundance protocol, which was performed using MetaBAT2 and the option “-m 1500” [31].

The quality of MAGs was estimated using the lineage-wf workflow of CheckM (v1.1.3) [32] and cmseq (https://github.com/SegataLab/cmseq), to classify MAGs as high-quality MAGs (> 90% completeness, < 5% contamination, < 0.5% non-synonymous mutations), and medium quality MAGs (> 50 completeness, < 5% contamination) for further analysis.

### MAGs analysis

MAGs were assigned to taxonomy groups using the CAT/BAT pipeline v.5.1.2 [33] and CAT_prepare_20200618 database. Those MAGs assigned to the bacterial genera *Lactococcus*, *Lactobacillus*, *Tetragenococcus*, *Staphylococcus*, *Corynebacterium*, *Brevibacterium*, and *Yaniella* were taxonomically assigned at species level using as reference the available genomes from these genera on the NCBI (ftp://ftp.ncbi.nlm.nih.gov/genomes/), which were selected and downloaded using the download_genomes pipeline (https://github.com/JoseCoboDiaz/download_genomes). The MAGs and NCBI genomes for each selected genus were employed for the calculation of average nucleotide identity (ANI) values by using dRep v2.6.2 [34]. Taxonomic assignment at species level was done by manual inspection of ANI phylogenetic trees obtained by dRep software. Moreover, the *Mdb.txt* output file obtained from all ANI calculations was transformed into a distance matrix, which was employed for the construction of a phylogenetic tree plot by *ggtree* R-package, using the UPGMA clustering algorithm (*hclust* method = "average").

The functional annotation of MAGs with completeness higher than 90% was performed by using eggNOG-mapper v2.1.5 [35] and the MMseqs algorithm [36]. The “KEGG_ko” column from the eggNOG-mapper output was employed to build the count matrix of KOs per MAG, where the information of the three hierarchical levels of the KEGG database was added, and only those KOs belonging to *09100 Metabolism* were kept for further analysis.

Additionally, all *Tetragenococcus* spp. genomes available at the NCBI (on the 20th June 2022) were downloaded to build phylogenetic trees, using the previously explained pipeline by dRep and *ggtree*, in order to confirm that new species (or at least not whole genome sequenced species) were detected, and to compare *T. koreensis* and *T. halophilus* genomes with those obtained in the current study.

### Strain-level analysis through an assembly-free approach

A strain-level population genomics analysis was undertaken for species of special interest, according to the analysis of MAGs, using the StrainPhlAn pipeline [37, 38]. Prior to StrainPhlAn analysis, metaphlan2 [39] was run for each sample, using the paired option as input and the bowtie2out command to obtain the alignment files in.sam format. The sample2makers.py script was employed to obtain the markers files for each sample, and extract_markers.py was used to extract clade markers for each species of interest, for further StrainPhlan analysis, according to the software designers (https://github.com/biobakery/biobakery/wiki/strainphlan1), and using as reference the corresponding species reference genome from the NCBI (Table S6). Phylogenetic trees were plotted by *ggtree* R-package, using the UPGMA clustering algorithm (*hclust* method = "average") and the *RaxML_bestTree* output file from StrainPhlAn analysis.

### Detection of horizontal gene transfer (HGT) events

A custom blast database was built with all MAGs obtained. A blastn [40] analysis of the MAGs fasta files, containing all MAG sequences correctly labeled on the headers to discriminate to which MAGs each contig belonged, was performed against the MAGs database with a 99.9% identity cutoff. The blastn output was filtered to remove hits where query and subject belonged to the same genus and to remove duplicated hits. Hits with alignment length shorter than 500 bp were also discarded for further analysis. HGT sequences were extracted by bedtools using the query sequences position from the blastn output, and eggNOG-mapper v2.1.5 [35] was employed, as previously described for MAGs, to functionally characterize HGT sequences. Detection of CDS related to integrons, transposases, or phages was done by searching “integron,” “transposase,” and “phage” on the eggNOG-mapper output file, while detection of plasmids was done by searching “relaxase” and by employing PlasFlow (https://github.com/smaegol/PlasFlow) as a second approach. The clustering of HGT sequences was done using VSEARCH [41] and 0.99 as identity cutoff. Chord diagrams for HGT events were plotted using the circlize R package.

### Diversity and taxonomy plots and statistical analysis

Only those taxa belonging to the kingdoms Bacteria and Fungi were used for further analyses, which were executed in parallel.

The *β*-diversity was estimated by principal component analysis using Bray–Curtis dissimilarity and the *vegdist* function. Within-group dispersion was evaluated through the *betadisper* function. Both functions are located in the R package *vegan* (https://github.com/vegandevs/vegan). The effects of cave, producer, and sample type (which includes ripening stage and cheese part for cheese samples and food contact *vs* nonfood contact surfaces for environmental samples) on sample dissimilarities were determined by permutational multivariate analysis of variance using distance matrices (PERMANOVA) with the *adonis* function in the R package *vegan* (https://github.com/vegandevs/vegan). The *compare_means* function in the R package *ggpubr* was used to include statistically significant differences on boxplot figures, which were plotted by using the R package *ggplot2* (https://github.com/tidyverse/ggplot2).

Comparisons between multiple groups of samples for taxonomy were performed by using the Kruskal–Wallis test and the post hoc Wilcoxon signed-rank test. *p*-values were adjusted through the Benjamini and Hochberg method [42], and significance was established at *p* < 0.05. All analyses were carried out using R version 3.6.2 (https://cran.r-project.org/).

### Source-tracking analysis

Genera relative abundance matrices were employed to attribute the main sources of the cheese microbial populations by using SourceTracker2 [43] with the *–per_sink_feature_assignments* parameter. The source-to-sink genera contribution dataset was reorganized by using *in-house* scripts (https://github.com/JoseCoboDiaz/piecharts_tax_st2) to plot a matrix of pie charts that indicates the influence of each source to each sink (by the size of the pie chart) and the percent influence of each genera in each source-to-sink pair.

### Resistome analysis

Reads were analyzed by bowtie2 v2.3.4.1 [27] alignment versus the ResFinder database [44], while contigs were analyzed by blastn [40] versus the same database. Blast output files were filtered to keep only those hits with at least 80% of identity and gene coverage. Contigs harboring ARGs were taxonomically classified using Kraken2 v2.0.8-beta [29] with the custom database employed for taxonomic read analyses. Plasmidic contigs were detected by Platon [45] and integrons by integron_finder2 [46]. ARG detection within integrons was performed using the ARG-contig_mobilome_analysis pipeline (https://github.com/JoseCoboDiaz/ARG-contig_mobilome_analysis), and the plot was done by using gggenes R-package (https://github.com/wilkox/gggenes) and edited with InkScape software (https://inkscape.org/).

### Supplementary Information

**Additional file 1:** **Figure S1.** General scheme of the sampling approach and analyses performed on blue-veined PDO *Cabrales* cheeses. **Figure S2.** Taxonomic composition of blue-veined cheese samples. **Figure S3.** Fungal beta-dispersion analysis. **Figure S4.** Taxonomic composition of “Source” samples. **Figure S5** Taxonomical (fungi) source attribution of cheese samples calculated by SourceTracker2 software. **Figure S6.** MAGs distribution by sample type. **Figure S7.** Phylogenetic trees based on ANI distance (for MAGs) and StrainPhlan analysis. **Figure S8.** Species abundance. **Figure S9.** Phylogenetic trees for NCBI genomes and MAGs assigned to *T. halophilus* and *T. koreensis*. **Figure S10.** Functional differences on *Staphylococcus equorum* MAGs. **Figure S11.** PCoA plots for resistome analysis at read level.

**Additional file 2:** **Table S1.** Statistical analyses performed on cheese samples from Cave2 at the final ripening stage (Stage3), for detecting differences between producers. **Table S2.** Statistical analyses performed on cheese samples from Producer2 at the final ripening stage (Stage3), for detecting differences between caves. **Table S3.** Characteristics of the main HGT-clusters detected. **Table S4.** Blastn output for HGT sequences versus ResFinder database. **Table S5.** Fungal genomes added to the customized kraken2 database. **Table S6.** Reference genomes employed on phylogenetic trees built by StrainPhlan analysis.

## Data Availability

The raw reads fastq files from shotgun metagenome sequencing of the 133 samples and the genome fasta files from the 613 medium and high-quality MAGs obtained are available under NCBI BioProject ID PRJNA764004, with physical BioSample accession numbers from SAMN21465692 to SAMN21465824 and SRA accession numbers from SRR15927537 to SRR15927669 for raw reads fastq files, and BioSample accession numbers from SAMN23026855 to SAMN23027467 for MAGs-genome fasta files.

## Abbreviations

PDO: Protected designation of origin
MAGs: Metagenome-assembled genomes
HGT: Horizontal gene transfer
KO: KEGG Orthology
CDS: Coding regions
BPW: Buffered peptone water
PCA: Plate count agar
MRS: De Man-Rogosa-Sharpe
LAB: Lactic acid bacteria
OGYEA: Oxytetracycline glucose yeast extract agar
VRBGA: Violet red bile glucose agar
CFU: Colony-forming units
NCBI: National Center for Biotechnology Information
ANI: Average nucleotide identity
