# Supplementary material for: The detailed analysis of the microbiome and resistome of artisanal blue-veined cheeses provides evidence on sources and patterns of succession linked with quality and safety traits
Source: Microbiome. 2024 Apr 27;12:78. doi: 10.1186/s40168-024-01790-4 (PMC11055350; doi:10.1186/s40168-024-01790-4)
Supplement: Supplementary file 2 — Additional file 1: Figure S1. General scheme of the sampling approach and analyses performed on blue-veined PDO Cabrales cheeses. Figure S2. Taxonomic composition of blue-veined cheese samples. Figure S3. Fungal beta-dispersion analysis. Figure S4. Taxonomic composition of “Source” samples. Figure S5 Taxonomical (fungi) source attribution of cheese samples calculated by SourceTracker2 software. Figure S6. MAGs distribution by sample type. Figure S7. Phylogenetic trees based on ANI distance (for MAGs) and StrainPhlan analysis. Figure S8. Species abundance. Figure S9. Phylogenetic trees for NCBI genomes and MAGs assigned to T. halophilus and T. koreensis. Figure S10. Functional differences on Staphylococcus equorum MAGs. Figure S11. PCoA plots for resistome analysis at read level. [file 40168_2024_1790_MOESM1_ESM.docx]

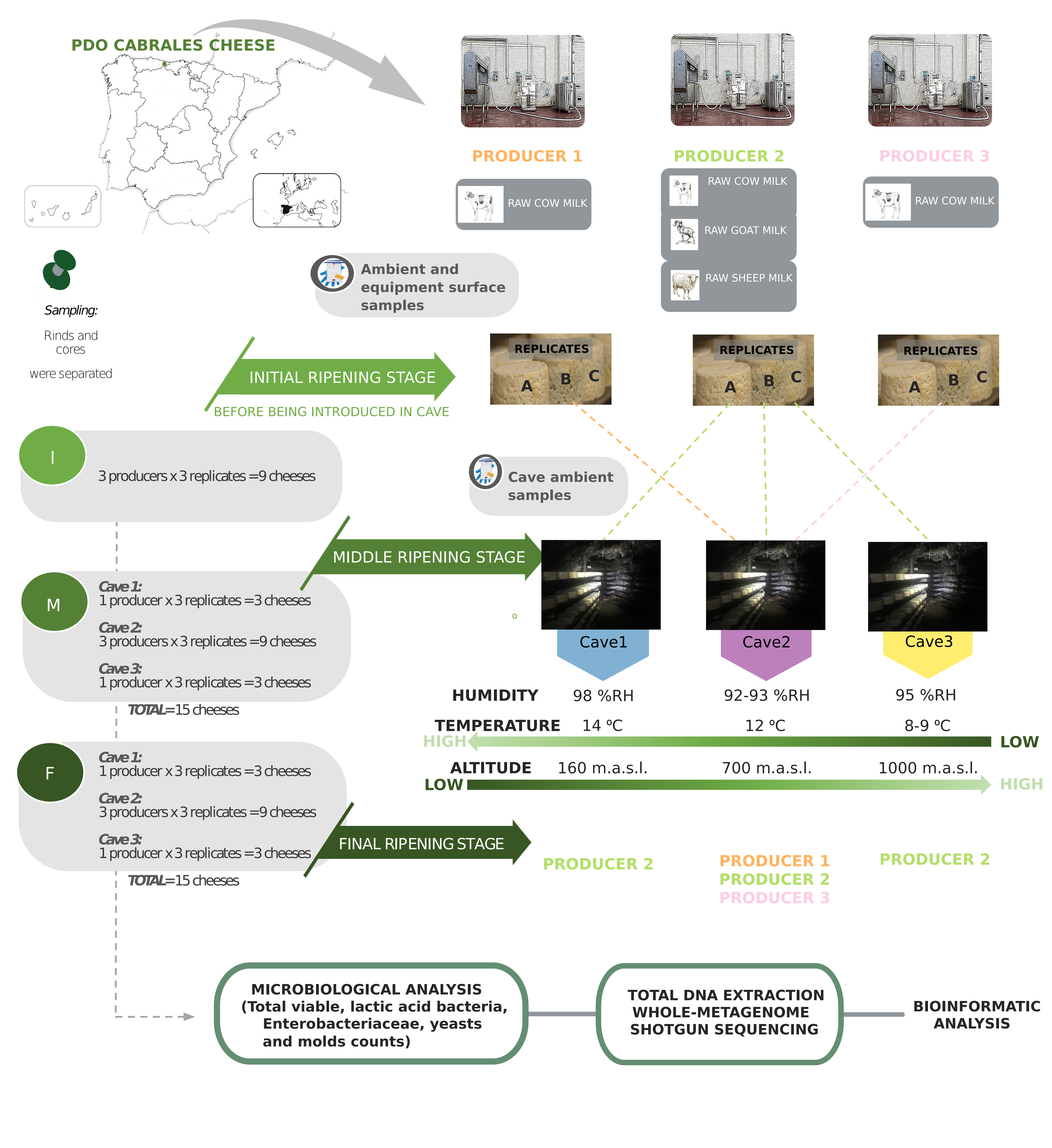
**Figure S1. General scheme of the sampling approach and analyses performed on blue-veined PDO *Cabrales* cheeses.**

**
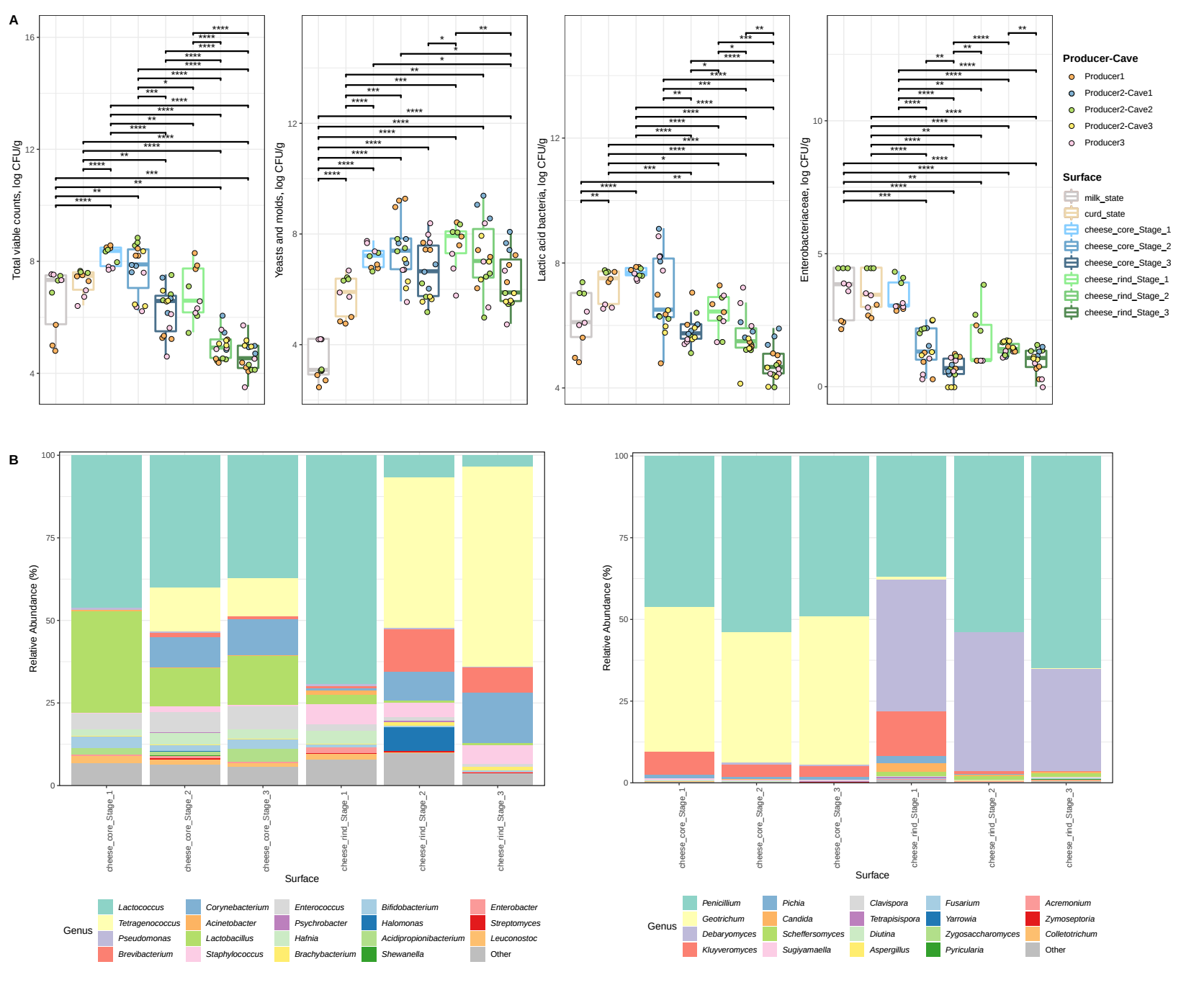
**

**Figure S2. Taxonomic composition of blue-veined cheese samples. (A)** Boxplot representation of the succession of microbial indicators, obtained by culture-dependent techniques, during the cheese-making and ripening process. The boxplots and assigned colors indicate the type of product sampled whereas colored dots inside the boxplot represent the cheese samples origin (producer and/ or cave). (**B)** Barplot representation of the relative abundance (%) of the main bacterial genera and fungal genera, obtained by whole metagenome shotgun sequencing, in cheeses along their ripening process. Each bar represents the average value for the cheese core or rind at each ripening stage for the 5 producer-cave groups analyzed.


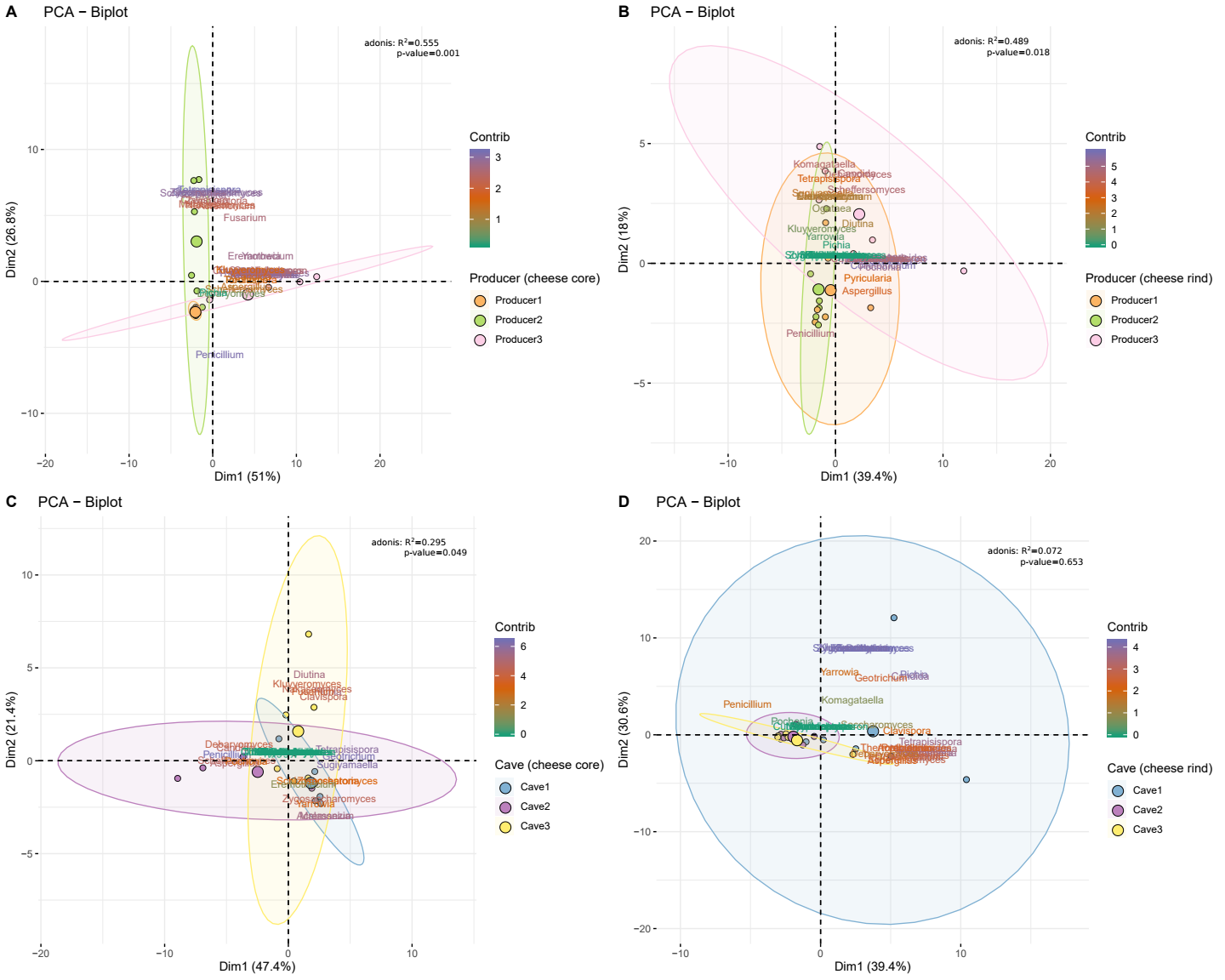


**Figure S3. Fungal beta-dispersion analysis.** Biplot of the principal component analysis of the forty most abundant fungal genera found in the cheese core **(A,C)** and cheese rind **(B,D)** samples from the 3 producers, ripened at Cave2 **(A,B)** and from Producer2 samples, ripened in the three different caves **(C,D)**. The biplots show the most significant fungal genera contributing to the cheese microbiome. Colored concentration ellipses (size determined by a 0.95-probability level) show the observations grouped by Producer or Cave.


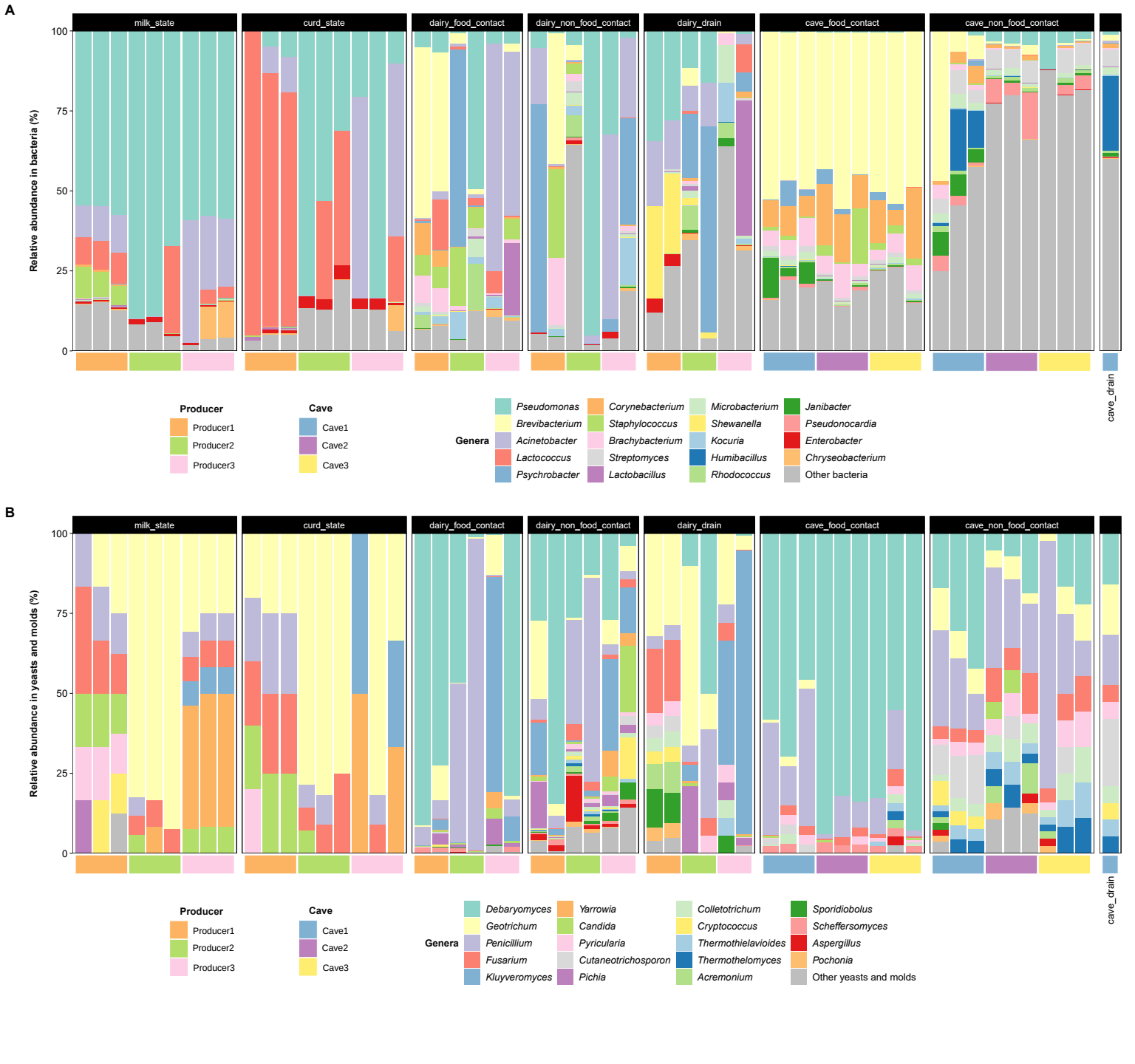
**Figure S4. Taxonomic composition of “Source” samples.** Barplots representing the relative abundance of the main bacterial **(A)** and fungal **(B)** genera from those samples considered as “source” in the SourceTracker analyses. Only the 19 most abundant genera were represented, while other genera were grouped as “Other”.

**
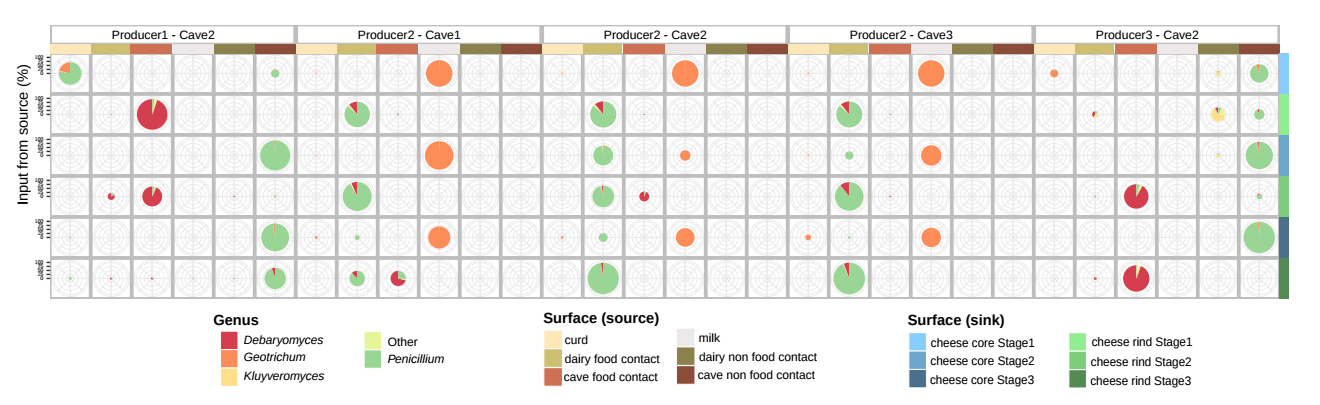
**

**Figure S5. Taxonomical (fungi) source attribution of cheese samples calculated by SourceTracker2 software.** Piechart plots represent fungal sources (on columns, *source* samples) for the cheese fungal community composition (on rows, *sink* samples). The ratio of the piechart is proportional to the percentage of *source* sample influence on *sink* sample (indicated on the y-axis). The colors within each piechart indicate the percentage of genus influence for each source-sink pair. Only *source* samples and the 4 main genera with significant influence were represented, while other genera were grouped as “Other”.

**
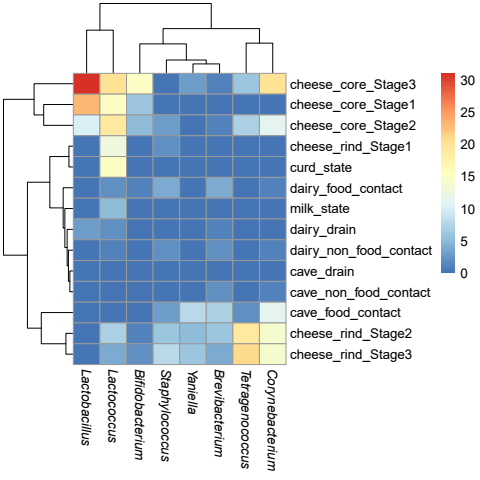
**

**Figure S6. MAGs distribution by sample type.** Pheatmap plot representing the amount of MAGs obtained for the main genera across the different sample types analysed.

**
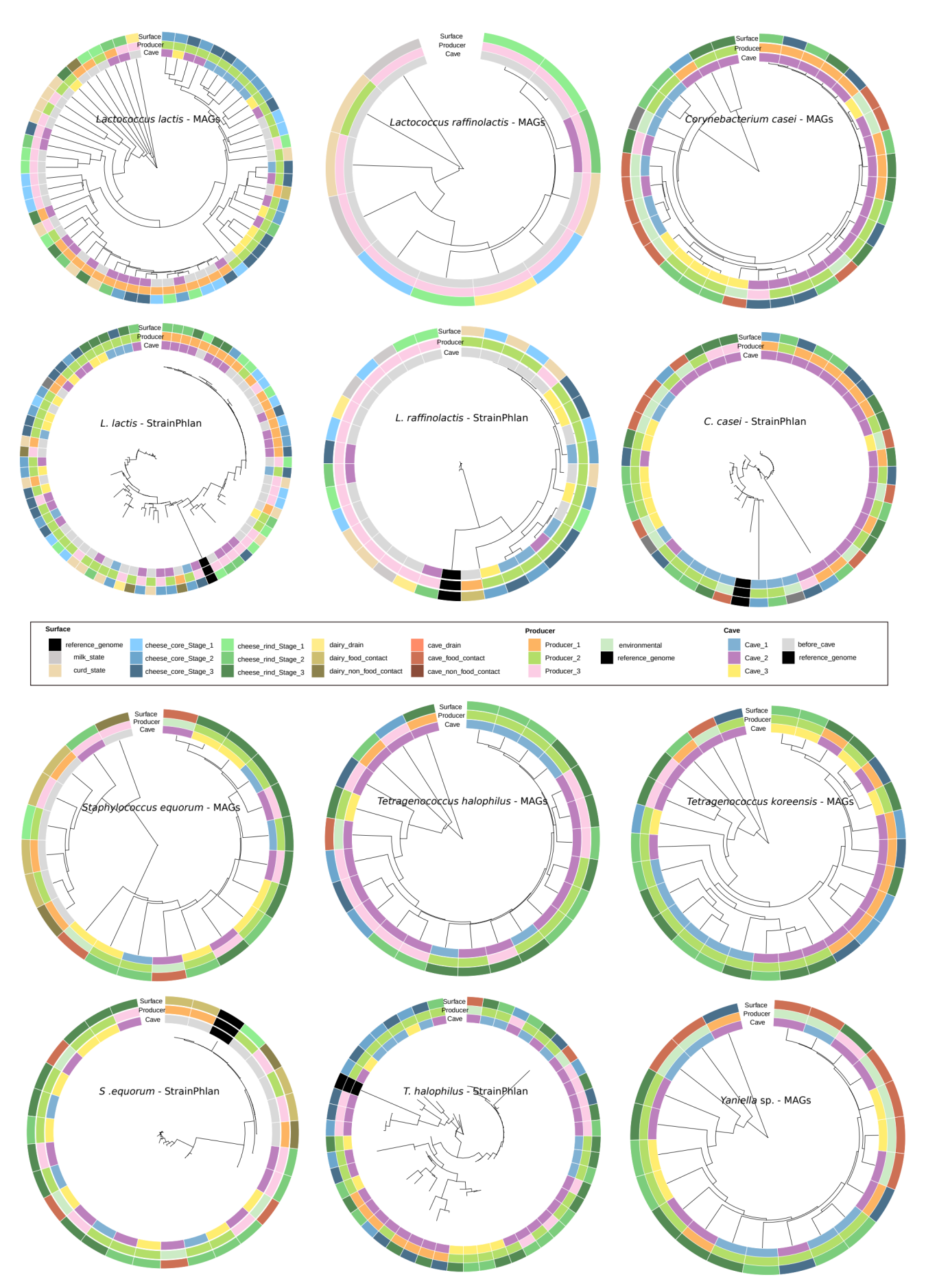
**

**Figure S7. Phylogenetic trees based on ANI distance (for MAGs) and StrainPhlan analysis.** *L. lactis*, *L. raffinolactis*, *C. casei*, *T. halophilus* and *S. equorum* were plotted using both apporaches, while, *T. koreensis* and *Yaniella* sp. were only plotted for MAGs, since *T. koreensis* and *Yaniella* sp. were not present on the StrainPhlan database.


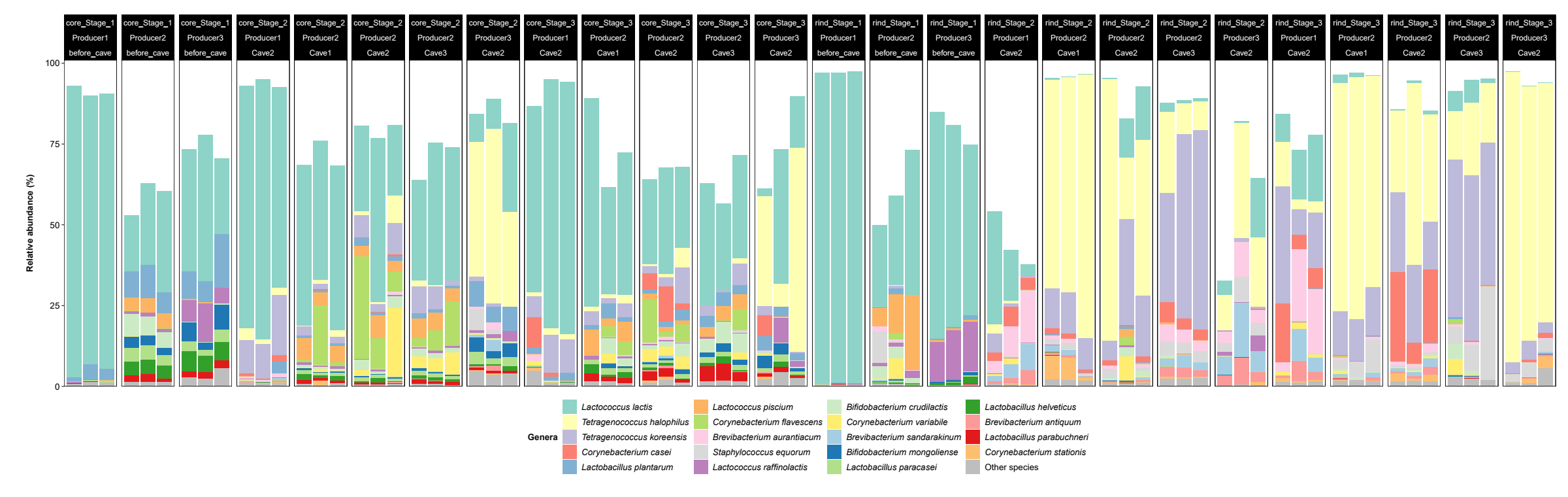
**Figure S8. Species abundance.** Barplot with the relative abundance (%) of the most relevant bacterial species across all sample types. Only those species not assigned as “Genus sp.” and belonging to genera of interest according to the MAGs characterisation results were represented. *Brevibacterium antiquum* and *Corynebacterium stationis* were not represented among MAGs. Species are sorted from highest to lowest abundance (average of all samples).

**
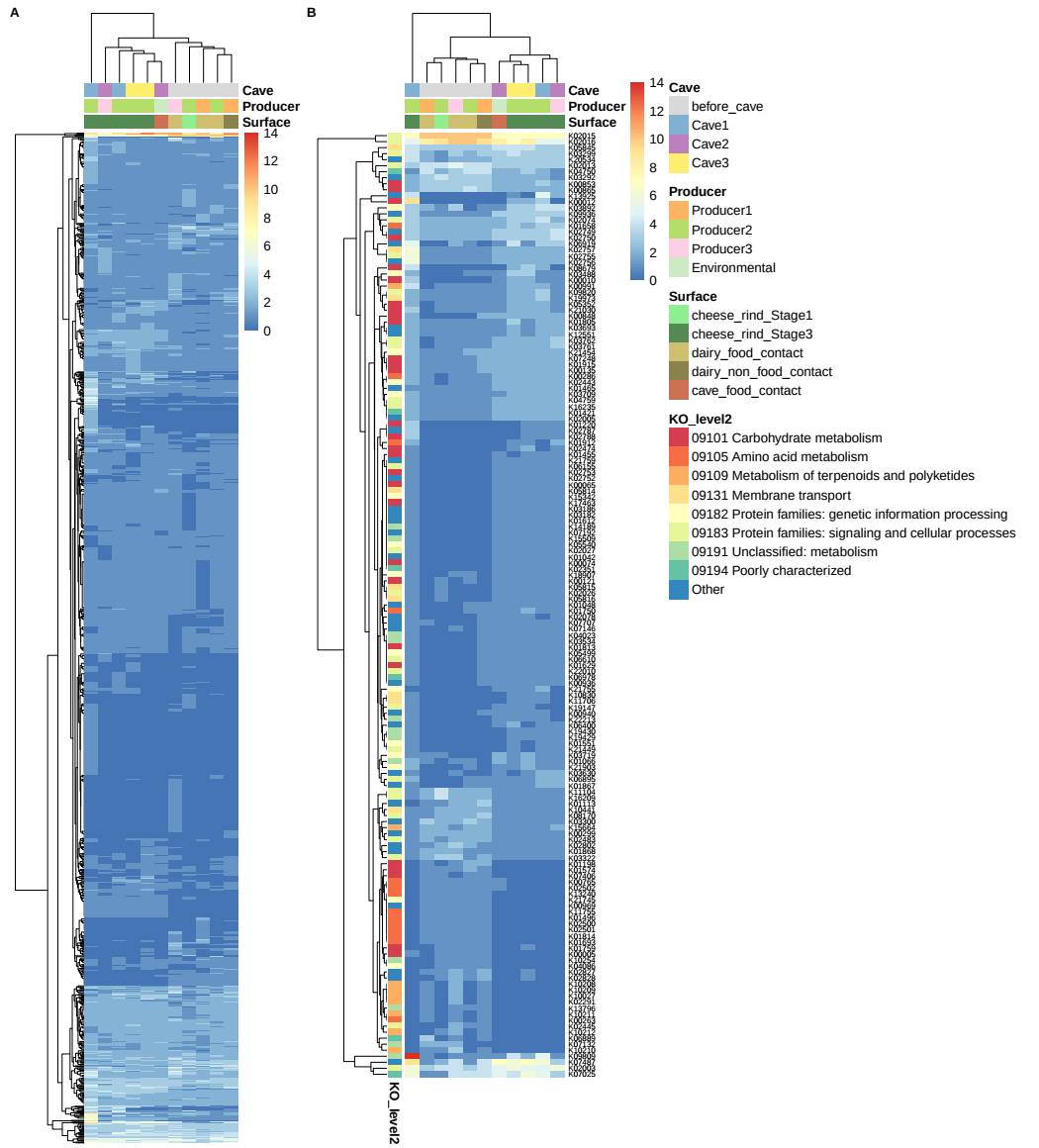
**

**Figure S9.** **Functional differences on *Staphylococcus equorum* MAGs.** Pheatmap representing the total CDS content (by KEGG Orthology code) **(A)** and those CDS with significant differences (Wilcoxon test, p-value<0.05) **(B)** between MAGs obtained from food processing environments within factories and cheeses at Stage1, and MAGs from cave environments and cheeses at Stage2 and Stage3.


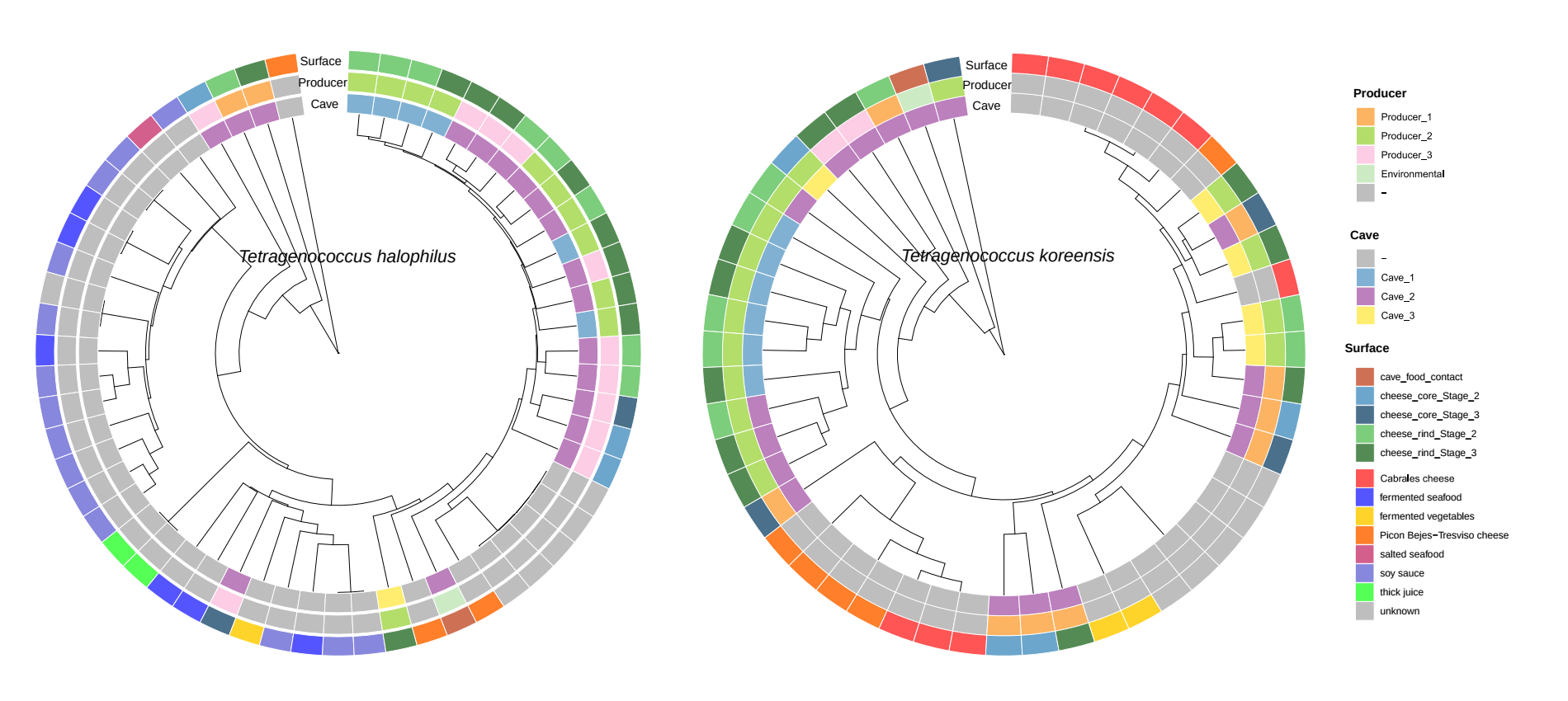
**Figure S10. Phylogenetic trees for NCBI genomes and MAGs assigned to *T. halophilus* and *T. koreensis*.** Performed by ANI distance for all *T. halophilus* and *T. koreensis* genomes available at the NCBI database together with the MAGs for these species obtained in the current study. Surface circle indicates surface (for MAGs) and strain isolation origin (for genomes downloaded from NCBI).


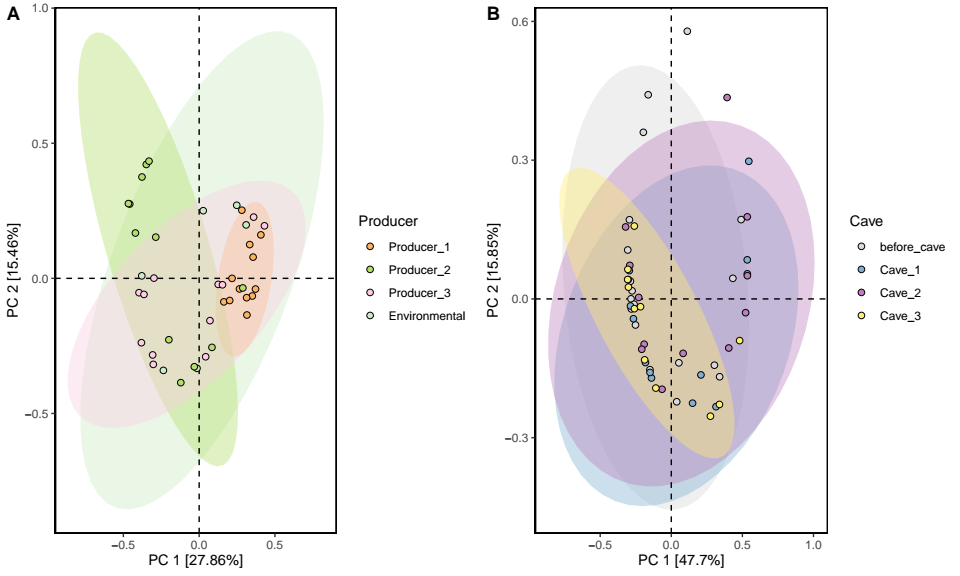
**Figure S11.** PCoA plots for resistome analysis at read level. **(A)** Samples from Cave2, comparing influence of Producer. **(B)** Samples from Producer2, comparing influence of Cave.
