# Supplementary material for: The detailed analysis of the microbiome and resistome of artisanal blue-veined cheeses provides evidence on sources and patterns of succession linked with quality and safety traits
Source: Microbiome. 2024 Apr 27;12:78. doi: 10.1186/s40168-024-01790-4 (PMC11055350; doi:10.1186/s40168-024-01790-4)
Supplement: Supplementary file 3 — Additional file 2: Table S1. Statistical analyses performed on cheese samples from Cave2 at the final ripening stage (Stage3), for detecting differences between producers. Table S2. Statistical analyses performed on cheese samples from Producer2 at the final ripening stage (Stage3), for detecting differences between caves. Table S3. Characteristics of the main HGT-clusters detected. Table S4. Blastn output for HGT sequences versus ResFinder database. Table S5. Fungal genomes added to the customized kraken2 database. Table S6. Reference genomes employed on phylogenetic trees built by StrainPhlan analysis. [file 40168_2024_1790_MOESM2_ESM.docx]

**Table S1. Statistical analyses performed on cheese samples from Cave2 at the final ripening stage (Stage3), for detecting differences between producers.** Only 10 most abundant (A) bacterail and (B) fungal genera are shown. The t-test and p-adjust method “BH” (Benjamini & Hochberg) were employed for pairwise analysis, and the Kruskal-Wallis test was employed for multiple global comparisons.

**A)**


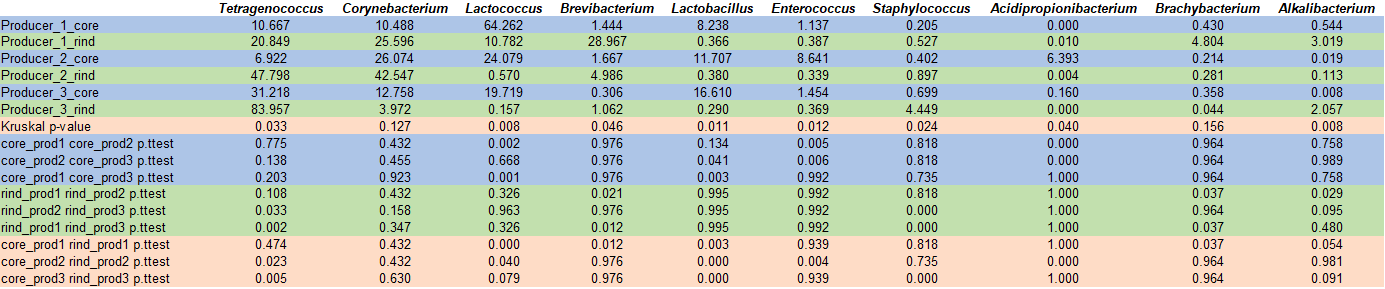
**B)**

**Table S2. Statistical analyses performed on cheese samples from Producer2 at the final ripening stage (Stage3), for detecting differences between caves.** Only 10 most abundant (A) bacterail and (B) fungal genera are shown. The t-test and p-adjust method “BH” (Benjamini & Hochberg) were employed for pairwise analysis, and the Kruskal-Wallis test was employed for multiple global comparisons.

**A)**

**B)**

**Table S3. Characteristics of the main HGT-clusters detected.** Only HGT-associated contigs longer than 20 kbases are shown. Plasmids were detected by Plasflow and by eggNOG output search for “relaxase” hits, while transposase, phage and integrase CDS were searched only on eggNOG outputs. Functions at Level 3 of the KEGG Orthology database are represented.

**Table S4. Blastn output for HGT sequences versus ResFinder database.** Only hits with %identity and %coverage > 80 were included.

**Table S5. Fungal genomes added to the customized kraken2 database.**

**Table S6. Reference genomes employed for the phylogenetic trees built by StrainPhlan analysis.**
